# Recent Advancements on Photothermal Conversion and Antibacterial Applications over MXenes-Based Materials

**DOI:** 10.1007/s40820-022-00901-w

**Published:** 2022-08-24

**Authors:** Shuyan Hao, Hecheng Han, Zhengyi Yang, Mengting Chen, Yanyan Jiang, Guixia Lu, Lun Dong, Hongling Wen, Hui Li, Jiurong Liu, Lili Wu, Zhou Wang, Fenglong Wang

**Affiliations:** 1 Key Laboratory for Liquid-Solid Structural Evolution and Processing of Materials Ministry of Education, Shandong University, Jinan, 250061 People’s Republic of China; 2 Department of Virology, School of Public Health, Shandong University, Jinan, 250012 People’s Republic of China; 3 Shenzhen Research Institute of Shandong University, A301 Virtual University Park in South District of Nanshan High-Tech Zone, Shenzhen, 518057 People’s Republic of China; 4 School of Civil Engineering, Qingdao University of Technology, Qingdao, 266033 People’s Republic of China; 5 Department of Breast Surgery, Qilu Hospital, Shandong University, Jinan, 250012 People’s Republic of China

**Keywords:** MXenes, Antibacterial mechanisms, Photothermal properties, Antibacterial applications

## Abstract

**Highlights:**

- Fabrication, characterizations and photothermal properties of MXenes are systematically described.
- Photothermal-derived antibacterial performances and mechanisms of MXenes-based materials are summarized and reviewed.
- Recent advances in the derivative applications relying on antibacterial properties of MXenes-based materials, including in vitro and in vivo sterilization, solar water evaporation and purification, and flexible antibacterial fabrics, are investigated.

**Abstract:**

The pernicious bacterial proliferation and emergence of super-resistant bacteria have already posed a great threat to public health, which drives researchers to develop antibiotic-free strategies to eradicate these fierce microbes. Although enormous achievements have already been achieved, it remains an arduous challenge to realize efficient sterilization to cut off the drug resistance generation. Recently, photothermal therapy (PTT) has emerged as a promising solution to efficiently damage the integrity of pathogenic bacteria based on hyperthermia beyond their tolerance. Until now, numerous photothermal agents have been studied for antimicrobial PTT. Among them, MXenes (a type of two-dimensional transition metal carbides or nitrides) are extensively investigated as one of the most promising candidates due to their high aspect ratio, atomic-thin thickness, excellent photothermal performance, low cytotoxicity, and ultrahigh dispersibility in aqueous systems. Besides, the enormous application scenarios using their antibacterial properties can be tailored via elaborated designs of MXenes-based materials. In this review, the synthetic approaches and textural properties of MXenes have been systematically presented first, and then the photothermal properties and sterilization mechanisms using MXenes-based materials are documented. Subsequently, recent progress in diverse fields making use of the photothermal and antibacterial performances of MXenes-based materials are well summarized to reveal the potential applications of these materials for various purposes, including in vitro and in vivo sterilization, solar water evaporation and purification, and flexible antibacterial fabrics. Last but not least, the current challenges and future perspectives are discussed to provide theoretical guidance for the fabrication of efficient antimicrobial systems using MXenes.
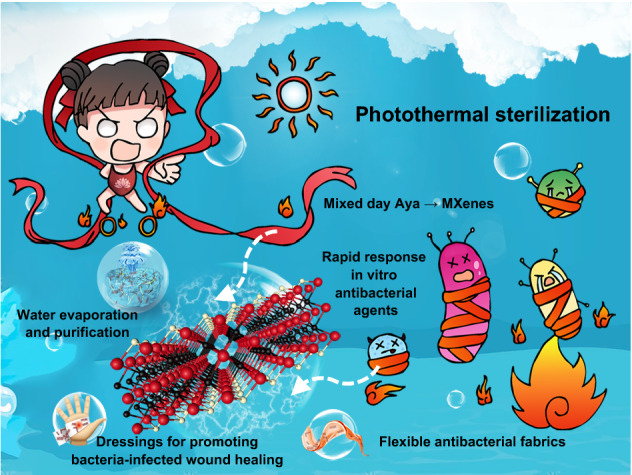

## Introduction

As a global problem, uncontrollable harmful bacterial proliferation has posed great threat to public health, which eventually caused economic burdens to the infected patients and the whole society [1, 2]. The infectious bacteria always brought in wound infections [3, 4], disease transmission [5, 6], water contamination [7], soil contamination [8], and widespread corrosions [9]. From a traditional viewpoint, antibiotics have held the top priority in the eradication of bacterial growth. Nevertheless, the emergence of antibiotic-resistant pathogens due to antibiotic abuse results in the failure of antibiotic therapy, becoming one of the most significant global challenges [10]. The drug-resistant bacteria have developed penetration barriers, drug efflux pumps, inactivating enzymes to protect themselves from the developed antibiotics [11, 12]. In addition, bacteria could also shelter from the attack of drugs or harsh conditions by forming the biofilm, a structure that serves as a sanctuary of the inner cells [13, 14]. Besides, horizontal gene transfer can disseminate resistance genes among biofilm wrapped bacteria, amplifying the bacterial resistance [15]. Unquestionably, the emergence of drug-resistant bacterial and the formation of biofilms leads to the dreadful biological crisis. Thus, searching for novel efficient treatment approaches without inducing drug resistance is of great significance for solving these issues. Recently, non-invasive photothermal therapy (PTT) functioning under light irradiation represents a promising alternative to antibiotics in the fight against bacterial proliferation [16, 17]. Based on hyperthermia beyond tolerance, this kind of therapy could efficiently damage the integrity of pathogenic bacteria in a controllable manner [18]. Furthermore, bacteria were unlikely to develop resistance to PTT as they did to antibiotics by drug excretion, facilitating metabolism and postponing absorption [11, 19]. In the PTT process, hyperthermia originating from the photo-to-thermal conversion process on the PTT agents could cause protein denaturation and inactivation, deoxyribonucleic acid (DNA) cross-linking and cell membrane loosening, which also facilitates the penetration of other available antibacterial agents to destroy the biofilm structure [20]. These satisfactory properties of PTT exhibit the feasibilities of using antibiotics-independent techniques to destroy harmful bacteria, thereby attracting extensive attention. To achieve desirable antibacterial efficiency and less light energy consumption, synergistic tactics by combining PTT with other techniques, such as photodynamic therapy (PDT) and metal ions incursion, have been brought into focus [21–23]. The heat generated by PTT could tremendously enhance the permeability of the bacteria membranes and promote the intracellular permeation of reactive oxygen species (ROS) or metal ions [24]. These synergistic tactics could not only lower the dosage requirements of photothermal agents, but also offset the imperfections of other therapies. In the post-antibiotic era, their high therapeutic efficiency has been proved, and it is considered safer and more thriving representatives in the field of antibacterial applications [21, 25, 26].

Various photothermal materials have been exploited and designed, such as carbon-based materials [27], noble metal nanoparticles [28], conjugated polymers [29], and metal–organic frameworks [30]. Among these candidates, MXenes have stood out due to the high aspect ratio, atomic-thin thickness, excellent photothermal performance, low toxicity, and ultrahigh dispersibility in aqueous systems [31–34]. MXenes represent a large group of two-dimensional (2D) transition metal carbides, carbonitrides, and nitrides with the general chemical formula M_n+1_X_n_T_x_ [33]. In this chemical formula, M represents early transition metals like Ti, Mn, V, Nb, Mo, Sc, Cr, etc., X stands for C and/or N, T_x_ means surface-terminating functional groups (mainly OH, O, and F), and n ranges from 1 to 5 [35]. This group of 2D materials exhibits strong light absorption performances of covering the entire ultraviolet (UV), visible, and near-infrared (NIR) ranges, showing great potential in photo energy conversion [36]. MXenes can directly act as PTAs or photosensitizers (PSs) by using photo-responsive building units, due to their intrinsic photothermal and photodynamic abilities [37, 38]. They can also achieve photo-induced medicinal ability by loading antibacterial agents or forming zero-dimensional (0D)/2D structures, one-dimensional (1D)/2D structures, and other architectures [39, 40]. Profoundly, the appropriate design and modification of MXenes could tune their light absorption performances [41], postpone the recombination of electrons and holes for promoting ROS generation [25], and improve the unique localized surface plasmonic resonance (LSPR) effect for enhanced photo-to-thermal conversion [42]. MXenes with low cytotoxicity and high biocompatibility are also excellent platforms for in vivo therapy, such as bacterial infection treatment, re-epithelialization acceleration, and granulation tissue formation, which vastly ameliorates the therapeutic efficacy with respect to traditional bandages [22, 27]. Moreover, profiting from their desired merits, including hydrophilicity, high aspect ratio morphology, and metallic conductivity, when MXenes are incorporated into thin film or hydrogel systems, they offer versatile potentials to design MXenes-based antibacterial soft materials with tunable properties [43, 44]. They also possess superb mechanical stability and solution processability, suitable for further assembly into various geometry and textile structures, thus realizing the concept of the recent trend in flexible and wearable electronic textiles for innovative antimicrobial applications [45, 46].

**Fig. 1:**
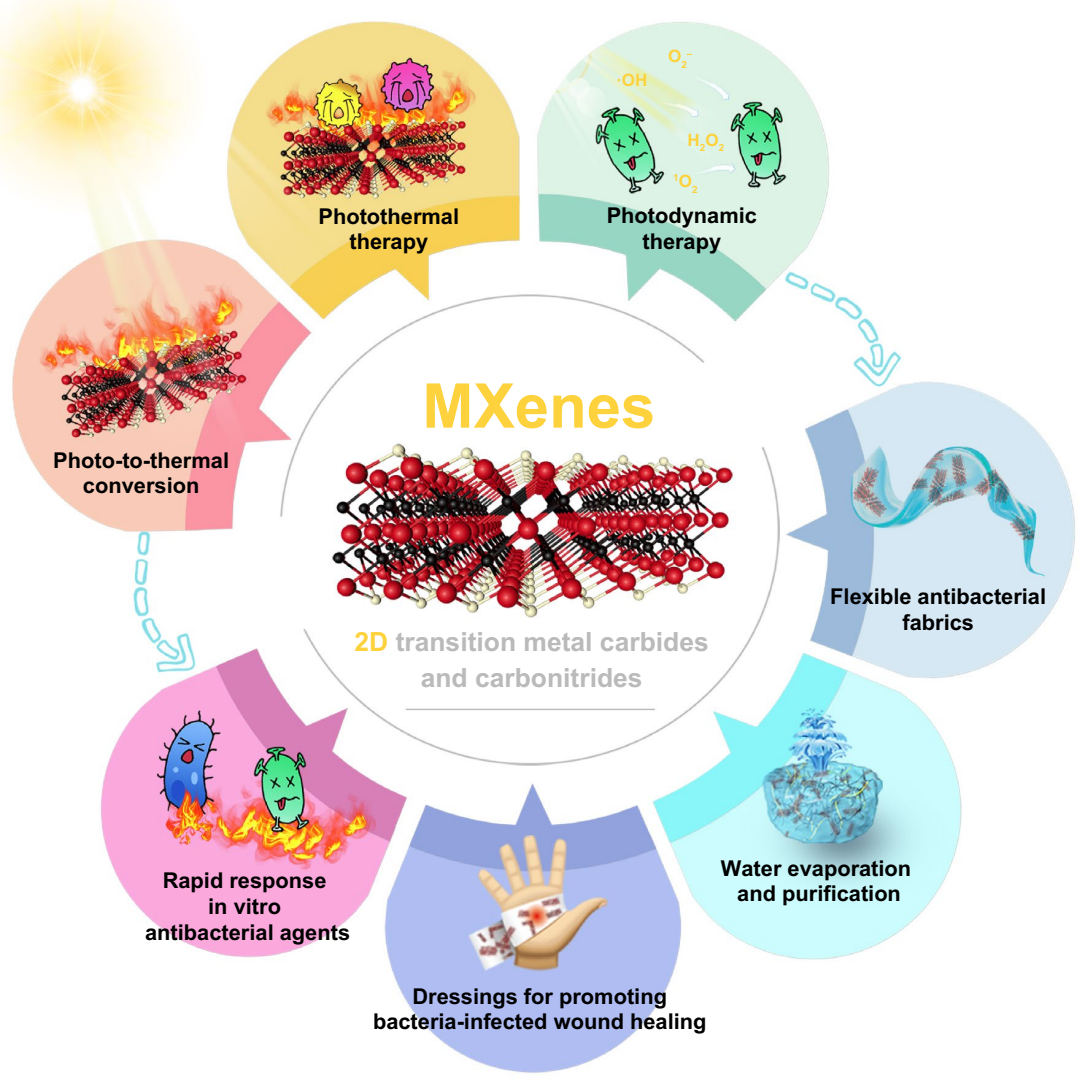
The effective photothermal antibacterial performances of MXenes endow them with widespread application potential

Vast amounts of research work have been dedicated in the past decade to designing MXenes-based antimicrobial materials displaying physical puncture, photothermal therapy and photothermal-derived therapy properties [28, 47, 48]. However, by searching the literature database, there have been no systematic review study summarizing the antimicrobial properties of photo-induced mechanisms, as well as research progress in photothermal antibacterial related applications. Herein, in this review, we firstly make a systematic summary and introduction to the synthetic process and textural properties of MXenes. Next, we comprehensively summarize and analyze the antibacterial mechanisms, mainly involving their inherent photo-to-thermal properties, photothermal therapy, and other photo-induced synergistic therapies. Based on these, current advances in their photothermal dominated sterilization are emphatically introduced, including rapid response in vitro antibacterial agents, dressings for promoting bacteria-infected wound healing, solar-driven water evaporation and purification, and flexible antibacterial fabrics (Fig. 1). Finally, we prospect the opportunities and challenges faced by MXenes-based materials for antibacterial applications in future.

## Synthesis and Microstructure of MXenes

MXenes, as a new family of 2D materials, have attracted research booms and achieved continuous progress since they came into sight. Here forward-looking summaries of the synthetic methodology and microstructure characterizations are presented. Comprehensive understandings of the preparation process and microstructure will deepen the fundamental knowledge of the properties of MXenes, which could enable their applications with ingenious contraptions in diverse emerging fields.

### Synthesis of MXenes

#### Approaches for Preparation of MXenes

**Fig. 2:**
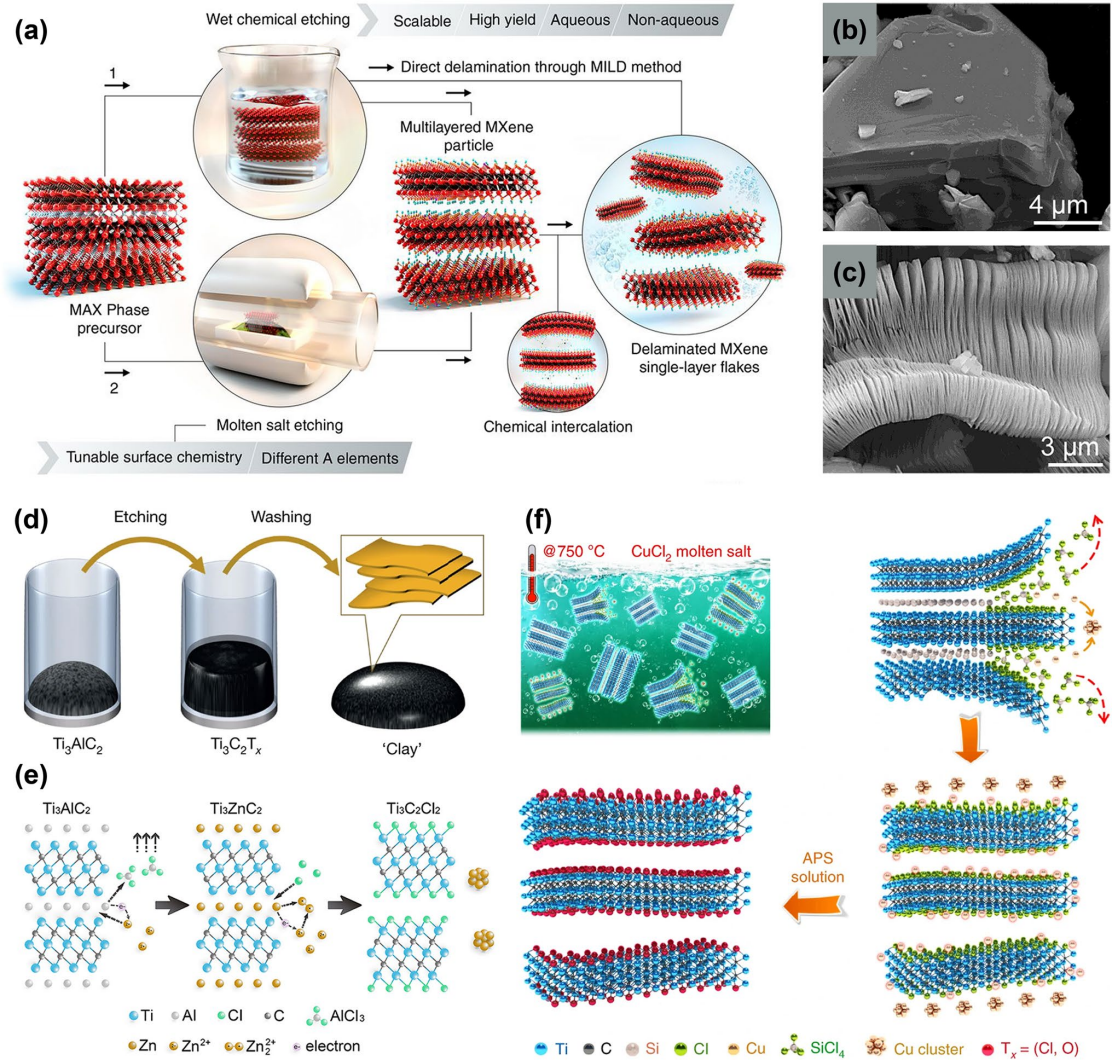
**a** Schematic illustration of two approaches to produce MXenes by removal of A layers from MAX phases and related layered compounds [35]. Copyright 2021, The American Association for the Advancement of Science. **b** SEM image of Ti_3_AlC_2_ particle before HF treatment; **c** SEM image of Ti_3_AlC_2_ particle after HF treatment [72]. Copyright 2012, American Chemical Society. **d** Schematic of clay-like MXenes etched by HCl and LiF [61]. Copyright 2014, Springer Nature. **e** Schematic of MXenes etched by molten ZnCl_2_ [66]. Copyright 2019, American Chemical Society. **f** Schematic of MXenes etched by molten CuCl_2_ [67]. Copyright 2020, Springer Nature

The MAX phase (M_n+1_AX_n_) is the precursor for the preparation of MXenes, in which the bonding between M and X shows the mixed characteristics of covalent bonds, metal bonds and ionic bonds, while the bonding between M and A exhibits the characteristics of metal bonds [49]. It can be seen that unlike graphene or molybdenum disulfide, in which the layers are combined through van der Waals forces, the MAX phase is more difficult to be exfoliated, and thus more corrosive agents are necessary [50]. For a typical synthetic approach, the preparation of MXenes proceeds in two steps, which are the selective etching of the A-layer atoms (e.g., Al, Si, Ga) from the corresponding MAX phase and the subsequent delamination of multilayered MXenes (Fig. 2a) [35]. After wet chemical etching, loosely stacked MX layers are obtained and can be further separated into single-layered flakes. Hydrofluoric acid (HF), as the etchant, was firstly employed to effectively eradicate the A layers from the MAX phase and generated OH and F surface terminated groups [51]. In a pioneering work, Naguib et al. [51] immersed the MAX phased powder in a 50 wt% hydrofluoric acid solution at room temperature and then washed the obtained suspension with deionized water. After etching and centrifugation, the original compact packing structure was converted into a slack accordion-like architecture, as shown in Fig. 2b, c. The following simplified reaction formula could describe the above process for selective removal of A layers from the MAX phase:

1 $$ {\text{Ti}}_{{3}} {\text{AlC}}_{{2}} {\text{ + 3HF}} \to {\text{AlF}}_{{3}} + \frac{3}{2}{\text{H}}_{2} + {\text{Ti}}_{{3}} {\text{C}}_{{2}} $$

2 $$ {\text{Ti}}_{{3}} {\text{C}}_{{2}} {\text{ + 2H}}_{{2}} {\text{O}} \to {\text{Ti}}_{{3}} {\text{C}}_{{2}} \left( {{\text{OH}}} \right)_{2} + {\text{H}}_{{2}} $$

3 $$ {\text{Ti}}_{{3}} {\text{C}}_{{2}} {\text{ + 2HF}} \to {\text{Ti}}_{{3}} {\text{C}}_{{2}} {\text{F}}_{{2}} + {\text{H}}_{{2}} $$

As a widely adopted method, hydrofluoric acid etching is effective for the rapid etching of the MAX phase. However, it cannot be ignored that HF solutions are highly corrosive, harmful and prone to cause over-etching [52]. Hence, in later stage, new etching systems such as HCl/LiF [53], NH_4_HF_2_ [54], and NH_4_F [55] have been employed to pursue mild etching conditions, which greatly enriched the synthetic approaches of MXenes. Incorporating cations (such as Li^+^, Na^+^, and Sn^4+^) between the M_n+1_X_n_ layers could lead to the expansion of the interlayer separations and the weakening of interlayer connections [56–60]. Therefore, MXenes nanosheets exhibit fewer defects, larger sizes, and more uniform thicknesses. For instance, Ghidiu et al. [61] added 1.98 g of LiF to 6 M HCl to etch Ti_3_AlC_2_ powders and successfully fabricated clay-like MXenes with excellent dispersibility and hydrophilicity (Fig. 2d). After being dried, the resulted hydrophilic materials could be shaped like clay and rolled into a thin film with thickness of a few micrometers. In addition to etching the common Ti_3_AlC_2_, Du et al. [59] found that after treating the Ti_3_AlCN MAX phase with the mixture of LiF and HCl, a uniform Ti_3_CNT_x_ colloidal solution could be obtained by ultrasonic and handshaking. With a “fluffy” morphology and a relatively small percentage of nanosheets, the Ti_3_CNT_x_ powder exhibited excellent charge storage and handling capabilities as well as excellent cycling properties. Apart from LiF/HCl system, NH_4_HF_2_ could also be used as an etchant by inserting the cation NH^4+^ in the accordion-like MXenes [62]. The etching process can be clearly illustrated in the following simplified reaction formula [63]. In particular, instead of AlF_3_, (NH_4_)_3_AlF_6_ as the product was produced, and among the layers of Ti_3_C_2_T_x_, NH_3_ and NH^4+^ were intercalated. In addition to the above procedures, researchers have also successfully synthesized MXenes through the reaction of other fluoride salts such as NaF and KF with hydrochloric acid [64, 65].

4 $$ {\text{Ti}}_{{3}} {\text{AlC}}_{{2}} {\text{ + 3NH}}_{{4}} {\text{HF}}_{{2}} \to \left( {{\text{NH}}_{{4}} } \right)_{3} {\text{AlF}}_{{6}} + {\text{Ti}}_{{3}} {\text{C}}_{{2}} { + }\frac{3}{2}{\text{H}}_{{2}} $$

5 $$ {\text{Ti}}_{{3}} {\text{C}}_{{2}} {\text{ + aNH}}_{{4}} {\text{HF}}_{{2}} {\text{ + 2H}}_{{2}} {\text{O}} \to \left( {{\text{NH}}_{{3}} } \right)_{{\text{c}}} \left( {{\text{NH}}_{{4}} } \right)_{{\text{d}}} {\text{Ti}}_{{3}} {\text{C}}_{{2}} \left( {{\text{OH}}} \right)_{{\text{x}}} {\text{F}}_{{\text{y}}} $$

Beyond that, current advances have demonstrated that MXenes could also be acquired through the relatively mild molten salt etching procedures without using the fluorine-containing materials [32]. One of them is the Lewis acid molten salt stripping method, which is much safer compared with the etching process. Li et al. [66] demonstrated that the MAX phase (Ti_3_AlC_2_) and molten ZnCl_2_ salt would react violently under specific conditions. In the reaction system, Zn^2+^ ions played a similar role as the hydrogen ion in HF, while Cl^−^ instead of F^−^ coordinated with M (Fig. 2e). As a result of the replacement process between the Zn element from molten ZnCl_2_ and the A-site element in MAX phase precursors (Ti_3_AlC_2_, Ti_2_AlN, and V_2_AlC), not only a series of new M_n+1_ZnX_n_ phases (Ti_3_ZnC_2_, Ti_2_ZnC, Ti_2_ZnN, and V_2_ZnC) were obtained, but also the MXenes with Cl-terminated functional groups (M_n+1_X_n_Cl_2_) were generated. Moreover, Li et al. [67] successfully extended the stripping strategy to a variety of Lewis acid chloride molten salts (such as CuCl_2_, ZnCl_2_, FeCl_2_, and AgCl) and broader MAX phase family members (such as the A-site element is Si, Al, Zn, and Ga). Figure 2f is the schematic illustration of Ti_3_C_2_T_x_ MXenes prepared by Ti_3_SiC_2_ MAX phase and CuCl_2_ Lewis molten salt. This study demonstrated that Cu^2+^ in molten salt could feasibly oxidize Si to Si^4+^ at 750 °C. Si^4+^ eventually formed SiCl_4_ with Cl^−^ and escaped from the Ti_3_C_2_ sublayer, while Cu^2+^ was reduced to Cu metal. The residual Cu species in the products could be removed using ammonium sulfate solution, and finally, Ti_3_C_2_T_x_ MXenes was obtained. In contrast with conventional etching methods, Lewis acid molten salt stripping process could proceed in a much more convenient and safer manner.

Besides the aforementioned top-down approaches through chemical etching and molten salt stripping methods, chemical vapor deposition (CVD) was recently studied as a bottom-up technique for the synthesis of MXenes. As compared to the top-down etching process, the material synthesized by CVD exhibits high crystalline feature [68]. The first demonstration of large-area ultrathin transition metal carbides (TMCs) through the CVD method was reported by Xu et al. [68]. In this study, methane was used as the carbon source, and Cu foil sat on a Mo foil as the substrate. At temperature higher than 1085 °C, high-quality 2D ultrathin TMCs crystals with a thickness of a few nanometers and a lateral dimension exceeding 100 μm were grown. Subsequently, Geng et al. [69] further extended this method to synthesize a high-quality and uniform Mo_2_C film from the initial micrometer range to the centimeter range on graphene. They also confirmed that graphene-templated growth of Mo_2_C exhibits a single crystal structure with a larger size and lower defect density. Compared with the MXenes obtained from etching methods, minor defects and no terminations in the CVD generated MXenes, open a new door for investigating their intrinsic properties and domain boundaries effect [70]. However, the structure generated by CVD is typically a thin film rather than a single layer, which is unsatisfied with the demands of mainstream applications, and thus more work should be done to achieve the CVD-produced single-layered MXenes in the future [71].

Table 1 summarizes the etching parameters of typical methods, including the etching method, MAX phases and their corresponding attainable MXenes, etchants, etching temperature, and etching time.

**Table 1:** Summary of typical methods used for the preparation of MXenes

Methods	Precursor	Obtained MXenes	Etching conditions		References
			Etchants	Time and Temp.^*a*^	
HF etching	Ti_3_AlC_2_	Ti_3_C_2_T_x_	HF (50 wt%)	2 h, RT^*b*^	[51]
	Ti_3_SiC_2_	Ti_3_C_2_T_x_	HF (30 wt%), H_2_O_2_ (35 wt%)	45 h, 40 °C	[73]
	V_2_AlC	V_2_CT_x_	HF (50 wt%)	90 h, RT^*b*^	[74]
	Nb_2_AlC	Nb_2_CT_x_	HF (50 wt%)	90 h, RT^*b*^	[74]
	Ti_3_AlCN	Ti_3_CNT_x_	HF (50 wt%)	18 h, RT^*b*^	[72]
	Mo_2_TiAlC_2_	Mo_2_TiC_2_T_x_	HF (50 wt%)	90 h, 55 °C	[75]
	Mo_2_Ti_2_AlC_3_	Mo_2_Ti_2_C_3_T_x_	HF (50 wt%)	48 h, RT^*b*^	[75]
	Mo_2_Ga_2_C_2_T_x_	Mo_2_C	HF (50 wt%)	160 h, 55 °C	[76]
In situ HF formation etching	Ti_3_AlC_2_	Ti_3_C_2_T_x_	HCl (6 M), LiF	45 h, 40 °C	[61]
	Ti_2_AlC	Ti_2_CT_x_	HCl (12 M), NaF (4 M)	24 h, 60 °C	[55]
	Ti_3_AlC_2_	Ti_3_C_2_T_x_	HCl (12 M), KF (4 M)	48 h, 40 °C	[55]
	Ti_3_AlC_2_	Ti_3_C_2_T_x_	HCl (12 M), NH_4_F (4 M)	48 h, 40 °C	[55]
	Ti_3_AlC_2_	Ti_3_C_2_T_x_	HCl (12 M), NH_4_F (4 M)	24 h, 30 °C	[55]
	Ti_3_AlC_2_	Ti_3_C_2_T_x_	NH_4_HF_2_ (2 M)	24 h, RT^*b*^	[52]
	V_2_AlC	V_2_CT_x_	HCl (12 M), NaF (2 g)	72 h, 90 °C	[77]
Lewis acid molten salt etching	Ti_3_SiC_2_	Ti_3_C_2_T_x_	CuCl_2_	24 h, 750 °C, Ar	[67]
	Ti_3_ZnC_2_	Ti_3_C_2_T_x_	FeCl_2_	24 h, 750 °C, Ar	[67]
	Ti_3_ZnC_2_	Ti_3_C_2_T_x_	CoCl_2_	24 h, 750 °C, Ar	[67]
	Ti_3_ZnC_2_	Ti_3_C_2_T_x_	NiCl_2_	24 h, 750 °C, Ar	[67]
	Ti_3_ZnC_2_	Ti_3_C_2_T_x_	CuCl_2_	24 h, 750 °C, Ar	[67]
	Ti_3_ZnC_2_	Ti_3_C_2_T_x_	AgCl_2_	24 h, 750 °C, Ar	[67]
	Ti_3_ZnC_2_	Ti_3_C_2_T_x_	CdCl_2_	24 h, 650 °C, Ar	[67]
	V_2_AlC	V_2_CT_x_	ZnCl_2_	5 h, 550 °C, Ar	[66]
	Ti_2_AlC	Ti_2_CT_x_	ZnCl_2_	5 h, 550 °C, Ar	[66]

^*a*^Temp.: temperature; ^*b*^RT: room temperature

#### Delamination of MXenes

**Fig. 3:**
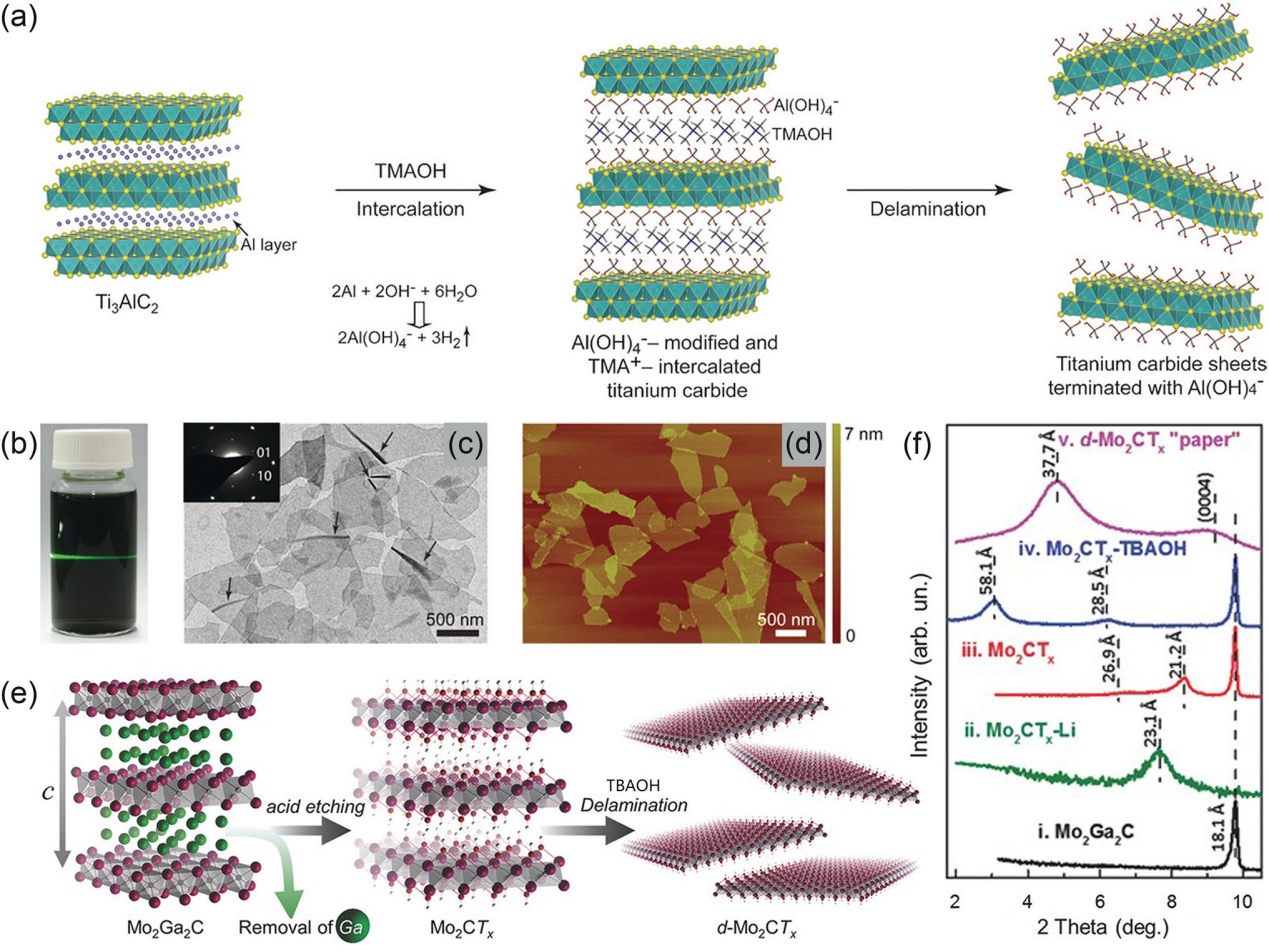
**a** Schematic illustration of the intercalation and delamination process; **b** Photograph of the obtained nanosheet dispersion in H_2_O with an apparent Tyndall effect; **c** Transmission electron microscopy (TEM) image of the extremely thin delaminated nanosheets; **d** AFM image of the extremely thin delaminated nanosheets [82]. Copyright 2016, Wiley–VCH. **e** Schematic illustration of the synthesis and delamination of Mo_2_CT_x_; **f** XRD patterns of Mo_2_Ga_2_C and Mo_2_CT_x_ [76]. Copyright 2016, Wiley–VCH

For the etched multilayered MXenes, delaminating them into 2D flakes with a few or even single layers is vital for better dispersibility, photothermal conversion ability, and high reactivity [78, 79]. 2D materials have long occupied an important status in photothermal conversion due to their robust dispersibility and facile surface functionalization [80]. When 2D nanomaterials come into service, their thickness is an essential factor affecting their photothermal properties. The thermal conductivity is also inclined to be higher for few-layered materials compared to multilayered materials [78]. Therefore, delamination (e.g., through sonication) is required to fabricate dispersed MXenes nanosheets, which increases the interlayer spacings and makes the layers exfoliated into the separated 2D feature [81]. It has been found that the use of intercalants can effectively facilitate the delamination of the multilayered MXenes. With the assistance of intercalants, the production of single/few-layered MXenes can be significantly increased by simple treatments such as handshaking, mechanical vibration and ultrasound sonication [49, 76]. For instance, by using tetramethylammonium hydroxide (TMAOH) as an intercalant, bulky TMA^+^ cations could effectively access the gallery space and facilitate delamination [82]. The subsequent breakdown of the precursor layered crystals into separated elementary layers could be proceeded with the insertion of TMA^+^ cations, as shown in Fig. 3a. In the obtained colloidal solution, the appearance is almost transparent, indicating multilayered MXenes were delaminated into extremely thin sheets (Fig. 3b, c). In the atomic force microscope (AFM) illustration, the height was estimated to be ca. 1.6–2.0 nm by line scanning across the plain area of the flakes (Fig. 3d). In this way, it was confirmed that in the colloidal solution, monolayer and bilayer nanosheets were dominated. Apart from TMAOH, Halim et al. [76] used tetrabutylammonium hydroxide (TBAOH) as the intercalant to delaminate Mo_2_CT_x_ under sonication in an ice-cold ultrasonic bath for one hour, realizing the synthesis and delamination of 2D Mo_2_CT_x_ (Fig. 3e). The XRD pattern showed that in the absence of TBAOH treatment, two peaks ascribed to (0002) facets appeared corresponding to c lattice parameters (c-LPs) of 21.2 and 26.9 Å, respectively (Fig. 3f). Upon intercalation with TBAOH, two (0002) peaks appeared corresponding to c-LPs of 28.5 and 58.1 Å, which means the intercalation of TBA^+^ cations and water molecules.

Many kinds of intercalants such as metal ions, organic molecules, and inorganic molecules have been used to delaminate the multilayered MXenes and increase the yield of single/few-layered MXenes [33, 82–84]. Due to the increasing application fields, MXenes nanosheets with high quality and low contents of defects are highly desired, and it is far-reaching to review the previous studies to select the proper experimental conditions properly. The selection of intercalants and the design of intercalation conditions significantly influence defect rate, particle size, specific surface area, surface properties, and yield [33, 52, 83]. Table 2 summarizes the intercalation conditions used for different precursors under various etching conditions.

**Table 2:** Summary of intercalation conditions used for the preparation of MXenes through etching methods

Precursor	Obtained MXenes	Etching conditions		Intercalation conditions	References
		Etchants	Time and Temp.^*a*^		
Ti_3_AlC_2_	Ti_3_C_2_T_x_	HCl (6 M), LiF	45 h, 40 °C	Sonication, 1 h	[61]
Ti_3_AlC_2_	Ti_3_C_2_T_x_	HF (50 wt%)	18 h, RT^*b*^	Dimethyl sulfoxide, 18 h, RT; Sonication, 4 h	[85]
Ti_3_AlC_2_	Ti_3_C_2_T_x_	HCl (37.2 wt%), LiF	24 h, 35 °C	TBAOH, 2 h	[86]
Ti_3_AlC_2_	Ti_3_C_2_T_x_	HF (50 wt%)	22 h, RT	Dimethyl sulfoxide, 18 h, RT; Sonication, 6 h	[83]
Ti_3_AlC_2_	Ti_3_C_2_T_x_	HF (50 wt%)	18 h, RT	N_2_H_4_·H_2_O, 24 h, RT	[87]
Ti_3_AlC_2_	Ti_3_C_2_T_x_	HF (40 wt%)	4 h, 40 °C	NaOH, 2 h, RT	[88]
Ti_3_AlC_2_	Ti_3_C_2_T_x_	HF (0.1 g mL^−1^)	0.5 h, RT	TMAOH, Handshaking, 2 min, RT	[82]
Ti_3_AlC_2_	Ti_3_C_2_T_x_	HF (49 wt%)	20 h, 60 °C	Sonication, 5 h	[89]
Ti_3_AlC_2_	Ti_3_C_2_T_x_	HCl (9 M), LiF	24 h, 35 °C	Sonication, 1 h	[90]
Ti_2_AlC	Ti_2_CT_x_	HF (10 wt%)	10 h, ice bath	Dimethyl sulfoxide, 18 h, RT; Sonication, 4 h	[91]
Mo_2_Ga_2_C	Mo_2_CT_x_	HF (14 M)	160 h, 55 °C	TBAOH, 18 h, RT	[76]
V_2_AlC	V_2_CT_x_	HF (48 wt%)	92 h, RT	TBAOH, (C_4_H_9_)_4_NOH, 2–21 h, RT	[84]

^*a*^Temp.: temperature; ^*b*^RT: room temperature

### Microstructure and Surface Properties of MXenes

**Fig. 4:**
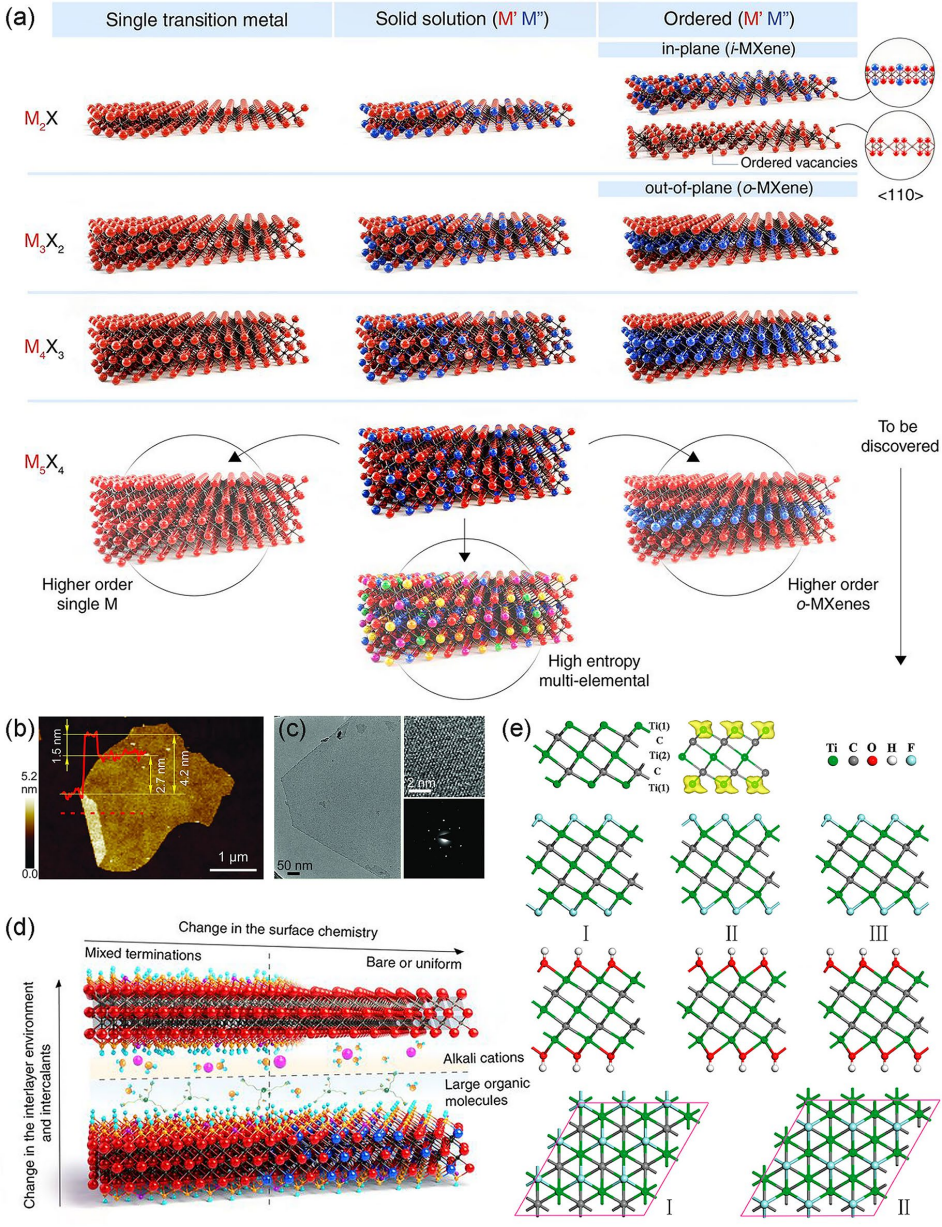
**a** Schematic illustration of the MXenes with a general formula of M_n+1_X_n_T_x_ [35]. Copyright 2021, The American Association for the Advancement of Science. **b** The AFM image of a folded Ti_3_C_2_T_x_ flake on Si/SiO_2_; **c** The HRTEM image and SAED image of Ti_3_C_2_T_x_ [93]. Copyright 2016, Wiley–VCH. **d** Schematic illustration of the surface of MXenes is covered with various terminations [35]. Copyright 2021, The American Association for the Advancement of Science. **e** Schematic illustration of the optimized geometries of the free-standing Ti_3_C_2_ monolayer and its fluorinated and hydroxylated structural forms [102]. Copyright 2012, American Chemical Society

The characteristic microstructure of MXenes determines their unique physical and chemical properties. Similar to its parent phase (M_n+1_AX_n_), MXenes with the general formula M_n+1_X_n_T_x_ exhibit hexagonal close-packed crystal structure [33]. Here, the early transition metal atoms at the M site follow the hexagonal close-packed model, and the carbon or nitrogen atoms at the X site fill the octahedral voids [33]. In MXenes, carbon and nitrogen atoms randomly fill octahedral voids, independent of carbonitride stoichiometry [92]. As shown in Fig. 4a, the M_2_X, M_3_X_2_, M_4_X_3_, and M_5_X_4_ are the most common formula of the currently discovered MXenes [35]. In M_3_C_2_ and M_4_C_3_ materials, M atoms follow the face-centered cubic packing structure, but in the M_2_X structure, the M atoms adopt the hexagonal close packing modes. The recently obtained M_5_C_4_ further enriched the diversity of the structure and expanded the theoretical number of MXenes to more than 100 [35]. At the microscopic level, the delaminated MXenes exhibit a typical lamellar structure. This morphology can be revealed by AFM micrographs (Fig. 4b) [93]. The layer spacing of MXenes has been experimentally verified to be 1 to 1.5 nm, and these values are wider than the reported graphene and phosphorene [61, 94, 95]. Furthermore, the hexagonal lattice structure in the axis zone along the c direction can be demonstrated from the high-resolution transmission electron microscopy (HRTEM) images, and the selected area electron diffraction (SAED) image confirmed the six-fold reflexes of lattice (Fig. 4c) [93].

Depending on the synthetic method used and the composition of MXenes, the surface of MXenes is covered with various terminations (Fig. 4d) [35]. Terminations are represented by T_x_, which can be O, OH, NH, F, Cl, Br, S, Se, Te [92, 96]. The reaction in fluorine- and chlorine-containing solutions results in MXenes with mixed surface terminations, whose composition can be expressed as (OH)_m_O_x_F_y_Cl_z_ [97–99]. Through thermal treatment and vacuum calcination, the terminations of MXenes can be modified in composition and coordination [100, 101]. For example, at temperatures above 775 °C, the terminations of Ti_3_C_2_T_x_ can be completely defluorinated [100]. Surface terminations are intractable to avoid in experiments, so understanding them is essential. As shown in Fig. 4e, previous work by Zhou et al. [102] probed the orientations of OH and F in Ti_3_C_2_X_2_. In type I, all F or OH groups are positioned above the hollow sites between three adjacent carbon atoms or point directly at the Ti atoms. For type II, all F or OH groups are found above the topmost C atoms on both sides of the main layer. The type III structure is a combination of types I and II, resulting in an asymmetrical arrangement on both sides of the main layer. In Ti_3_C_2_X_2_ configurations, the structural stability can be assessed by comparing their relative total energies. Among Ti_3_C_2_F_2_ and Ti_3_C_2_(OH)_2_, type I conformer is most favorable in energy. Even so, for these three types, structural relaxations of their monolayers maintain the original geometrical integrity. The functionalized MXenes are also thermodynamically stable as the Gibbs energy change required for their formation is negative [103]. Additionally, the modification of surface functional groups exhibits a promising design space through chemical modifications. For example, the Cl and Br groups on MXenes can be replaced by O, S, Se, and Te, creating unique structural and electronic properties [96].

## Photothermal Properties and Antibacterial Mechanisms of MXenes

Over the past decade, there have been plenty of studies concentrating on 2D materials for their bactericidal applications. The various antibacterial mechanisms of 2D materials, such as graphene (with physical insertion and chemical disruption) [104], MoS_2_ (with enhanced conjugation of bacterial/PTT), WS_2_ (with ROS release/damage the structural integrity of bacterial membrane), g-C_3_N_4_ (with photocatalytic self-cleaning), black phosphorus (with ROS release/membrane damage) have also been researched [105]. Intriguingly, rich tunability and promising new antibacterial strategies make MXenes stand out from numerous two-dimensional materials. Various advantages, including inherent 2D structure, satisfactory electromagnetic wave confinement and conversion capacity, and diverting LSPR effect, endow MXenes with satisfactory photo energy conversion ability, bringing in superb photo-induced antimicrobial effects [106]. The main target of photo-triggered antibacterial properties is to destroy bacterial cells in a particular way so as to perturb their life activity. Hence, the photothermal mechanisms of MXenes, mainly accounting for photo-induced antibacterial properties, are of tremendous importance and need to be described in detail. Interestingly, compared with sole PTT, various combined therapies with less light energy consumption show superior antibacterial potential and cost-effectiveness, achieving a desired synergistic effect [107, 108]. In this section, the photothermal properties of MXenes and their derivative antibacterial mechanisms will be systematically reviewed.

### Photothermal Mechanisms of MXenes

The photothermal conversion capability of nanomaterials refers to their ability to absorb certain light energy and convert it into thermal energy. The photothermal conversion mechanisms on materials are primarily determined by their inherent molecular or crystal structure, inter-particle coupling, intra-particle coupling, and electron distribution [109]. Generally speaking, 2D MXenes enjoy exceptional advantages in photo-to-thermal conversion owing to their inherent large absorption surface, abundant free electrons distribution, and strong absorption in broadband solar spectrum, etc. [110]. Nevertheless, the investigation of photothermal behavior over MXenes was initiated only a few years ago, and we must continue to admit that a deep understanding of the photothermal mechanisms is still not established. A slew of pioneering research has been executed to our knowledge with the purpose of unravelling the mechanisms.

**Fig. 5:**
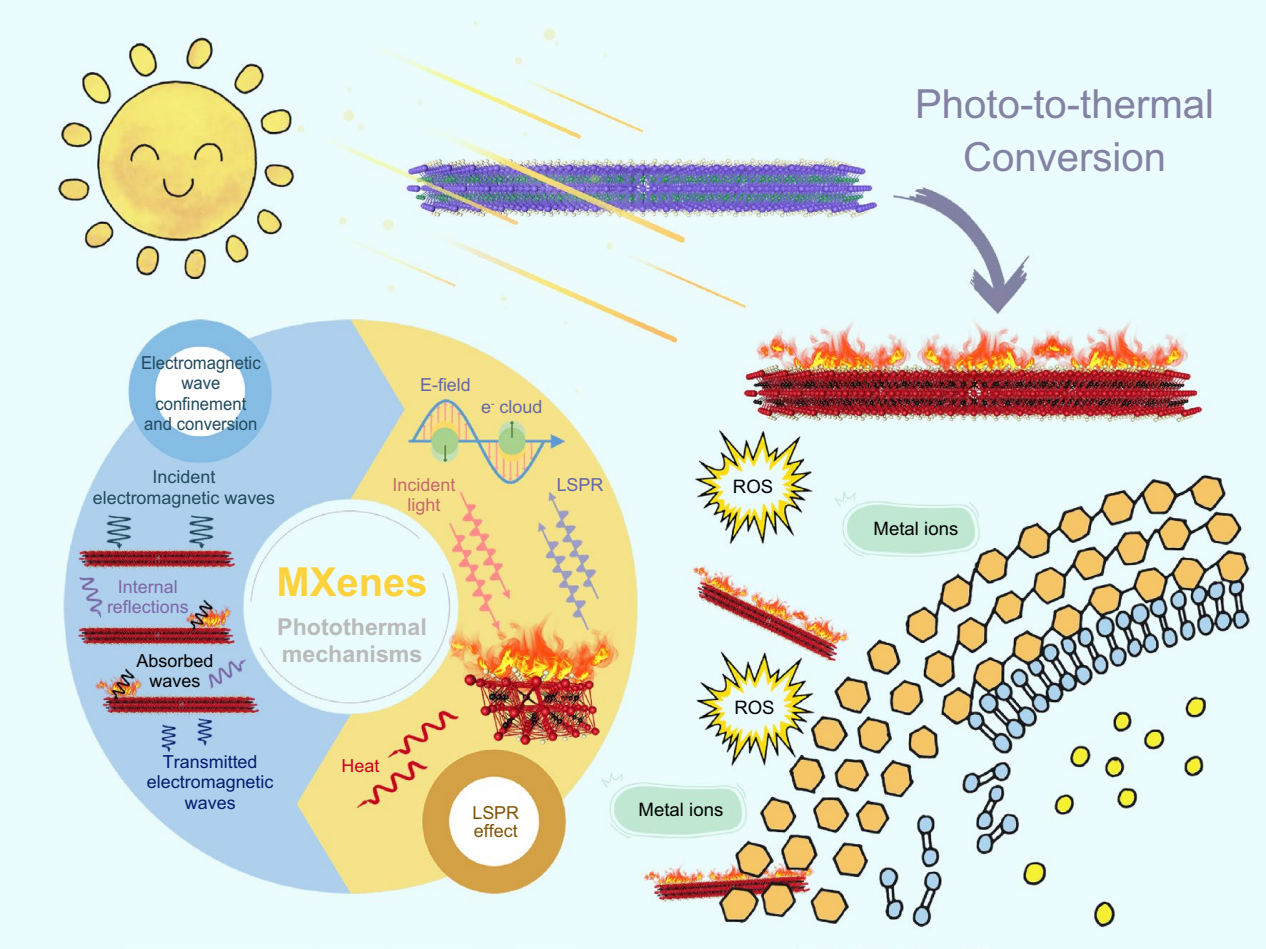
The schematic photothermal mechanisms of MXenes account for superb photo-induced antimicrobial effects

**Fig. 6:**
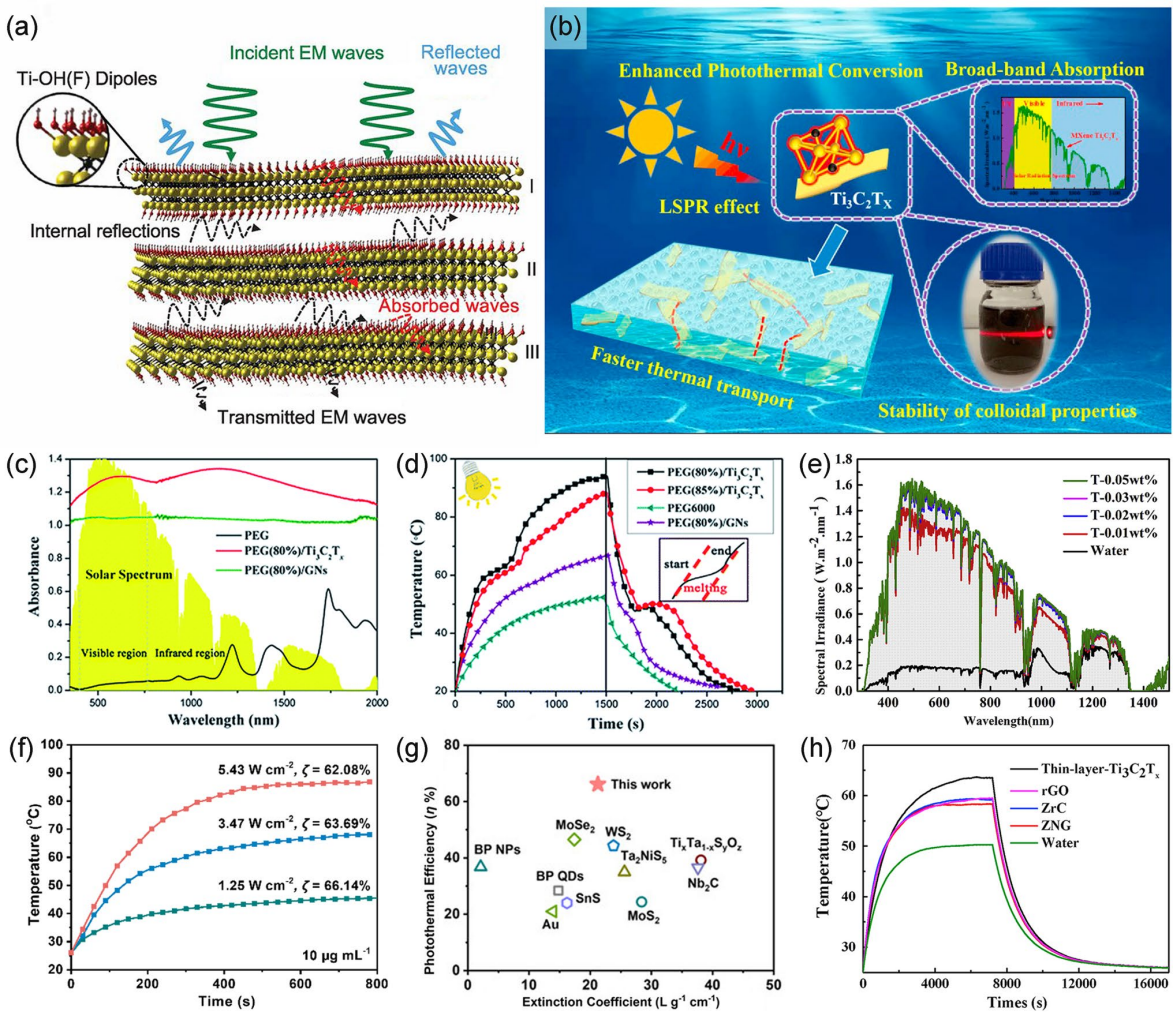
**a** Schematic illustration of electromagnetic wave penetration/reflection/absorption [86]. Copyright 2016, The American Association for the Advancement of Science. **b** Schematic illustration of the radiation energy conversion mechanism of Ti_3_C_2_T_x_ [106]. Copyright 2020, Elsevier. **c** Absorbance spectra of polyethylene glycol (PEG)/Ti_3_C_2_T_x_ composite; **d** Temperature evolution curves of the PEG/Ti_3_C_2_T_x_ composites under the simulated sunlight irradiation [111]. Copyright 2019, Royal Society of Chemistry. **e** The spectral irradiance of Ti_3_C_2_T_x_ with different concentrations [106]. Copyright 2020, Elsevier. **f** Photothermal response of Ti_3_C_2_T_x_ under 808 nm NIR light with various power; **g** Comparison between reported photothermal agents and Ti_3_C_2_T_x_ in terms of mass extinction coefficient and photothermal conversion efficiency [119]. Copyright 2021, Wiley–VCH. **h** The temperature of nanofluids containing different nanoparticles with the same mass fraction (0.02 wt%) [106]. Copyright 2020, Elsevier

(i) MXenes and MXenes-based materials can efficiently absorb light energy in virtue of their satisfactory electromagnetic wave absorption capacity, which is an indispensable prerequisite for photothermal behavior. Shahzad et al. [86] pioneered the discovery of multiple internal reflection behaviors in Ti_3_C_2_T_x_ flakes, which allowed them to dissipate and absorb the incident energy. On account of the high carrier concentration on the surface of MXenes, when electromagnetic waves reached the surface of the MXenes nanosheets, some waves would be reflected immediately. However, the induced local dipoles generated by the surface functional groups contributed to the absorption of incident light penetrating the Ti_3_C_2_T_x_ structure. A considerable part of the electromagnetic waves entered the lattice structure and reflected multiple times between layers. When transmitted waves with less energy encountered the next MXenes flake, the same process occurred, resulting in an overall attenuation. As depicted in Fig. 6a, in this process of penetration, reflection, absorption, the energy of electromagnetic (EM) waves is gradually consumed and converted into heat [86].
(ii) The LSPR effect, through which surface carriers of metallic nanomaterials could be regulated to produce heat, also dominates the photothermal mechanisms of MXenes (Fig. 6b) [106, 111]. In general, metal nanoparticles exhibiting the LSPR effect are conducive to photothermal conversion [112, 113]. When light waves are incident on the interface between the metal and the dielectric components, the free electrons on the metal surface oscillate collectively [114]. The light waves couple with free electrons on the metal surface to form near-field electromagnetic waves propagating along the metal surface. If the oscillation frequencies of the electrons coincide with the frequencies of the incident light waves, resonance will occur, and the electromagnetic field will be confined to a small space on the metal surface and enhanced [115]. Intriguingly, Mauchamp et al. [116] demonstrated that Ti_3_C_2_T_x_ exhibited plasmonic feature. The MXenes inherited from the MAX phase with metal-like characteristics possess a “semimetal” property [110]. Surface plasmons in MXenes are substantially dependent on the free charge carrier density on the surface, as reported for metals, semimetals, and semiconductors [117]. In this context, M_n+1_X_n_T_x_ is known to exhibit an evident metal-like free electron density, which is closely related to the abundant surface-terminated moieties (T_x_) [118]. Through spatially resolved ultra-high-resolution analysis, the longitudinal and transversal surface plasmson modes and the inherent interband transition sustained by flakes of MXenes could be unambiguously verified [117]. Profoundly, each monolayer in an MXenes flake behaves like an isolated sheet, sustaining a unique set of surface plasmon modes, revealing the particular 2D structure of MXenes and their fundamental divergence from “traditional” plasmonic metals [117]. Particularly in the visible and near-infrared ranges, MXenes exhibit longitudinal and transversal surface plasmon modes [35]. For example, two enhanced absorption peaks were observed in the absorbance spectra of Ti_3_C_2_T_x_ composite at 610 and 1148 nm, which was caused by LSPR strongly enhancing the light-matter interaction (Fig. 6c) [111]. These two enhanced absorption bands in the visible and NIR regions allow the Ti_3_C_2_T_x_ nanosheets to achieve a rapid sunlight-harvesting and photothermal conversion. Figure 6d displays the real-time dependence of temperature change, which also represented the switchable photothermal conversion performance [111]. Furthermore, it was reported that with the increase of the dispersion concentration of MXenes in the solvent, the corresponding absorption intensity and spectral irradiance were also increased [106]. Based on the advances in understanding the photo-to-thermal conversion mechanisms over MXenes, we have plotted the following figure to clearly illustrate this process (Fig. 5). Moreover, heat induced by PTT could boost the permeability of the bacterial cell membrane and speed up the cellular penetration of metal ions and ROS produced by PSs, thus achieving a desired synergistic treatment mode.

As a rule, the absorbed energy can be quantified by measuring the area under the solar spectral irradiance curve. MXenes possess plasmonic peaks covering the entire visible and near-infrared spectral region, and they also exhibit intense absorption in the ultraviolet range on account of interband transitions. As can be seen in Fig. 6e, the curve nearly overlapped with the spectral solar irradiance when the mass fraction of Ti_3_C_2_T_x_ was 0.05 wt%, indicating a broadband absorption ability in 200–1500 nm wavelength [106]. Therefore, most of the radiant energy was absorbed and directly converted into heat. In practical measurements, the photothermal conversion of MXenes when exposed to various lights is confirmed. The highest reported internal photothermal conversion efficiency of Ti_3_C_2_T_x_ could reach a shocking value of 100% [81]. Under the irradiation of 808 nm NIR light, the temperature of Ti_3_C_2_T_x_ suspension with a low concentration of 10 μg mL^−1^ quickly reached 90 °C within 500–600 s at a power density of 5.43 W cm^−2^ (Fig. 6f) [119]. Meanwhile, the suspension also exhibited stable recurrent photothermal response to several light on–off cycles, even under NIR irradiation with high power density, which indicated their acceptable reusability and stability. The photothermal conversion efficiency of Ti_3_C_2_T_x_ measured under 808 nm NIR light is superior to other reported photothermal materials, such as Cu_9_S_5_ (25.7%), Prussian Blue (41.4%), Au nanorods (21%), and nanovesicles (37%) (Fig. 6g) [119]. When 0.02 wt% Ti_3_C_2_T_x_ nanosheets were exposed to simulated sunlight irradiation with the energy intensity of 0.1 W cm^−2^, the maximum temperature rise was higher than other studied materials, including graphitic carbon (ZNG), ZrC, and reduced graphene oxide (rGO), as depicted in Fig. 6h [106]. The satisfactory photothermal conversion property ensures MXenes stand out from numerous 2D materials, promoting their potential in antibacterial applications where the temperature is one of the determining factors.

### Photothermal and Photothermal-derived Antibacterial Mechanisms over MXenes

#### Photothermal Therapy over MXenes

**Fig. 7:**
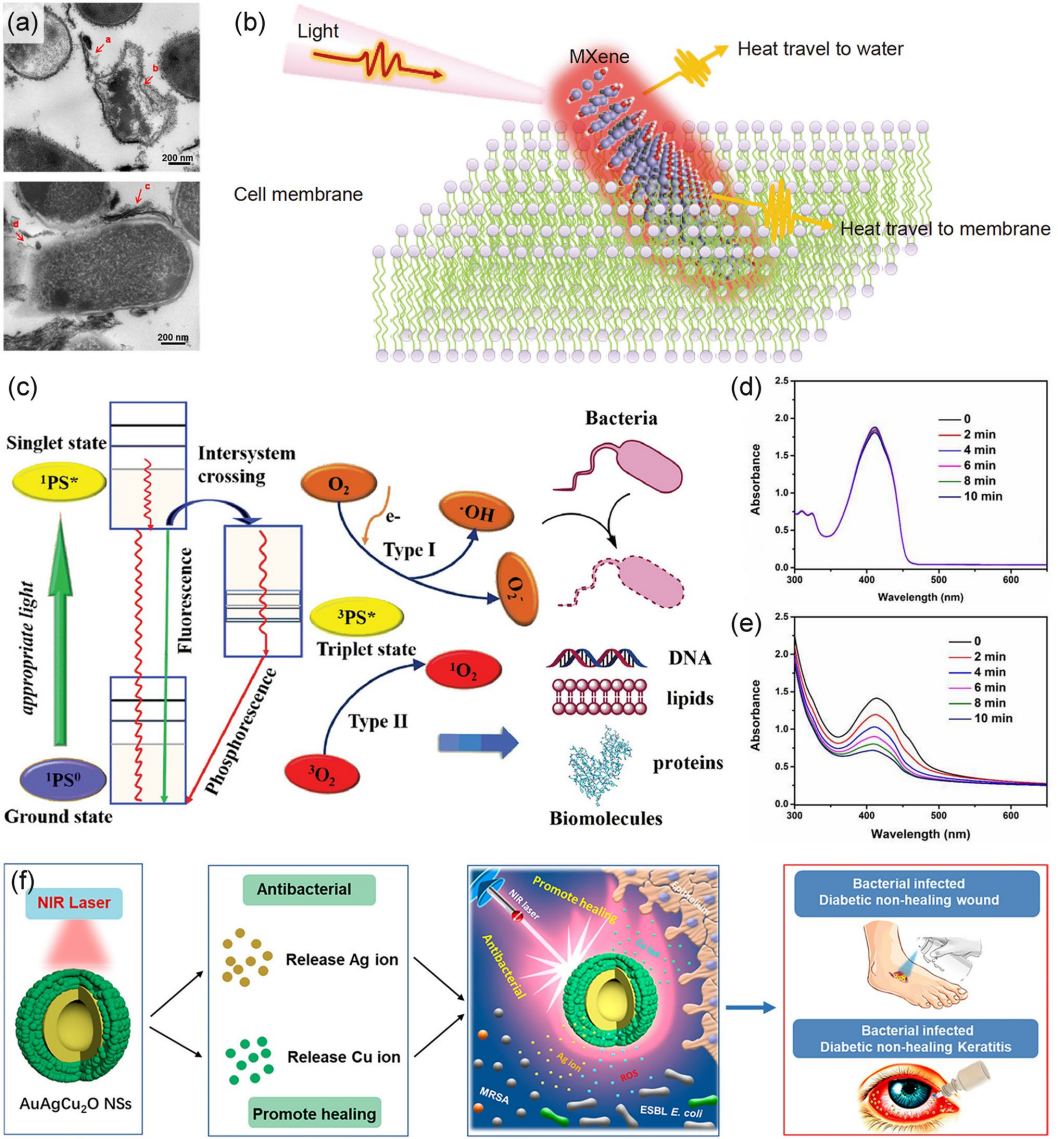
**a** TEM images of diverse bacteria treated with 200 μg mL^−1^ of Ti_3_C_2_T_x_ for 4 h [121]. Copyright 2016, American Chemical Society. **b** Schematic illustration of antibacterial mechanisms of MXenes with photothermal effect [37]. Copyright 2021, Springer Nature. **c** Photodynamic therapy mechanism of PSs under light irradiation [135]. Copyright 2019 Wiley–VCH. **d** Detection of DPBF’s singlet oxygen generation upon 808 nm NIR light irradiation; **e** Detection of singlet oxygen generation over Ti_3_C_2_T_x_ upon 808 nm NIR light irradiation [38]. Copyright 2017, American Chemical Society. **f** NIR-activated Ag ions and Cu ions kill bacteria and improve wound healing [108]. Copyright 2020, American Chemical Society

The effect of heat on bacteria has been widely studied. In general, the cellular structures and substances affected by heat are the cytomembrane, nucleoid, peptidoglycan cell wall, ribonucleic acid (RNA), ribosomes, and diverse enzymes [120]. The lethality of heat is based on the destruction of at least one pivotal component beyond a critical threshold, which results in the inhibition of life activity of the bacteria [120]. PATs with high photo-to-thermal conversion efficiency heat up under light irradiation, which seriously affects the life activities of surrounding pathogens. Moreover, MXenes can be unquestionably used as “nanothermal blades” owing to their excellent photothermal properties and inherently sharp 2D structures (Fig. 7a) [121]. Moreover, 2D flakes could be prepared into quantum dots, sequentially gaining various advantages, including high aqueous dispersibility, chemical stability, excellent optical property and easy functionalization [122–124]. Highly dispersed MXenes quantum dots exhibited whopping extinction coefficient and ultrahigh photothermal conversion efficiency, working as lethal “flocking nanothermal blades” to pathogenic cells [125].

Upon light irradiation, MXenes will effectively absorb and convert the light energy into heat, leading to the dramatic temperature rise on their surfaces, and this process will accelerate the death of the surrounding bacteria (Fig. 7b) [37]. The cell membrane, composed primarily of proteins, lipids, and polysaccharides, is significantly affected by heat generated on MXenes. Some studies have shown that membrane damage is linked to cell inactivation because of its partial loss of functionality, and the resulted imbalance of intracellular homeostasis has been demonstrated: disorganization in the entrance and outflow of several components [126, 127], suffocation of respiration activity [127] and loss of pH homeostasis [128]. The high temperature could also remarkably promote the permeability of pathogenic microorganisms’ membranes, thus enhancing ROS or metal ions with antibacterial ability to infiltrate (this section will be discussed in detail next).

DNA is an essential molecule for bacterial survival, and its direct implication in the inactivation is undoubted. Upon exposure to heat, mutation frequency would increase in surviving populations, revealing that heat treatment gives rise to the irreversible damage of DNA [129]. Proteins exist in bacterial cells, either as structural proteins or as enzymes. Relevant research has demonstrated that hyperthermia would also cause protein denaturation and aggregation in bacterial cells when the temperature rises to an unbearable level [130, 131]. Heat-induced protein denaturation could lead to functional loss in a variety of ways. Detoxifying enzymes, such as DNA repair enzymes, chaperones, proteases, and superoxide dismutase, which play a significant role in the self-regulation mechanism, are proven highly sensitive to heat [120]. Therefore, massive proteins will lose their function in bacterial cells if they are already thermally injured. Bacteria that have undergone photothermal treatment are almost impossible to develop antibiotic resistance by facilitating metabolism and reducing absorption [11].

Considering that MXenes exhibited high photothermal conversion capacity under light irradiation [23, 132, 133], the light-triggered antibacterial processes of MXenes could be inferred as follows: MXenes with sharp edges are feasible to adhere to or insert into pathogenic cells, and at the same time, irradiation energy of light absorbed by MXenes nanosheets significantly increases the temperature of MXenes, as a result of which the generated hyperthermia facilitates the ablation of bacterial structures, resulting in pathogenic bacteria death. Undoubtedly, PTT has become a well-trusted antibacterial therapy due to antibiotics-independent performance and selective hyperthermal treatment.

#### Photothermal/Photodynamic Synergistic Therapy and Other Photothermal- derived Therapy

Nevertheless, sole PTT cannot always meet the demand for antimicrobial applications. For example, higher temperatures exceeding the tolerance limits of healthy cells might be required if PTT is exclusively used to eradicate biofilms or drug-resistant bacteria, which greatly limits its potential in vivo applications [2]. Synergistic therapies, such as PTT/PDT and PTT/metal ions incursion, have emerged and received extensive attention in recent years as new strategies to solve these problems. For the combined approaches between PTT and PDT, the generated heat through photothermal conversion could increase the permeability of cell membranes and thus enhance the penetration ability of ROS into cells [2]. Photo-induced ROS, such as singlet oxygen (^1^O_2_), super oxide anions (O_2_^−^), and hydroxyl radical, has been widely acknowledged as powerful weapons to cause oxidative stress and sequentially sabotage the integrity of cytomembranes [26, 134]. Photodynamic therapy employs photo-responsive substances to generate ROS, which could further oxidize the surrounding biomolecules such as nucleic acids, lipids, and proteins to cause devastating damage to the target cells (Fig. 7c) [135]. Based on the ROS formation pathways, PDT is categorized into two types, namely, type I and type II [2, 135]. The PSs used in PDT are basically in the ground state (^1^PS^0^) and become excited to a singlet state (^1^PS^*^) after being irradiated with a specific light source. Through intersystem crossing, the electron transitions from the short-lived singlet state to the long-lived triplet state (^3^PS^*^). In type I process, ^3^PS^*^ transfers one electron directly to the adjacent substrate, generating free radicals or radical ions, mainly including O_2_^−^ and •OH. O_2_^−^ is an essential intermediate in the biosystem for generating H_2_O_2_ employing dismutation in the presence of superoxide dismutase or by one-electron reduction. Ulteriorly, the highly cytotoxic •OH can be obtained under the one-electron reduction of H_2_O_2_. Type I reaction occurs primarily on bacterial cell membranes, with unsaturated phospholipid molecules to extract hydrogen. These molecules further react with oxygen and form lipid peroxides, thereby disrupting structural integrity and making bacterial cell membranes more permeable. In the Type II process, the energy of the 3PS* is rapidly transferred to molecular oxygen (^3^O_2_) due to the electron spin multiplicity, leading to the excitation of ^3^O_2_ from the ground state to the excited singlet state, producing the so-called singlet oxygen (^1^O_2_) [136]. ^1^O_2_ is the most destructive ROS and can directly oxidize critical biomolecules in cells, such as lipids, peptides, and enzymes [137, 138]. The ROS generation ability of MXenes under irradiation could be evaluated by probes. For example, when the suspension of Ti_3_C_2_T_x_ nanosheets was irradiated with 808 nm NIR light, the absorbance of 1, 3-diphenylisobenzofuran (DPBF) as probe molecules was much lower than that of the control group without nanosheets, and the absorption intensity decreased with irradiation time increasing (Fig. 7d, e) [38]. The decrease in absorbance indicated that ^1^O_2_ was trapped by DPBF, verifying that ^1^O_2_ was generated from Ti_3_C_2_T_x_ nanosheets under NIR irradiation. Furthermore, the generation of ROS could be enhanced when MXenes were combined with other components, such as metal sulfides and metal oxide semiconductors, to form the nanocomposites [139, 140].

Bactericidal metal ions exhibited remarkable bactericidal effects by inactivating intracellular proteins and destroying bacterial membranes (Fig. 7f) [108]. These metal ions could noninvasively release and maintain their excellent bactericidal ability at low doses without exhibiting side effects when combined with PTT and PDT. For instance, Cu species could be anchored on the surface of MXenes in the form of sulfide or oxide, greatly improving the separation of the photo-generated electron–hole pairs upon irradiation, and as a result of this, enhanced ROS generation and accelerated penetration of copper ions can be expected [25, 139]. In general, by making full use of the physical puncture contribution of MXenes, the hyperthermia of PTT, and the facilitated penetration of ROS or metal ions, MXenes-based materials could bring in rapid destruction of the cell membranes as well as the accelerated collapse of the cellular homeostasis.

## Applications of MXenes in Antibacterial and Related Fields

As discussed in the previous section, MXenes-based materials have been regarded as a well-reliable antibacterial candidate for their remarkable photo-to-thermal conversion capacity as well as other photothermal-derived synergistic therapies potential. Multifunctional photo-responsive MXenes-based materials that integrate the advantages of photothermal effect and antibacterial activity are increasingly used in many fields. In this section, we summarize the state-of-the-art achievements utilizing MXenes-based materials for in vitro/vivo clinic trials, water purification and smart fabrics where bacteria-killing is necessary.

### In Vitro Antibacterial Applications

In the last decade, the application prospect of MXenes in antibacterial has been exhaustively confirmed and rapidly developed. However, pure MXenes cannot always meet the demand for photothermal sterilization. Hence, synergistic therapies such as MXenes/other antimicrobial agents and MXenes-based photodynamic hybrids have been regarded as new countermeasures.

#### Pure MXenes

**Fig. 8:**
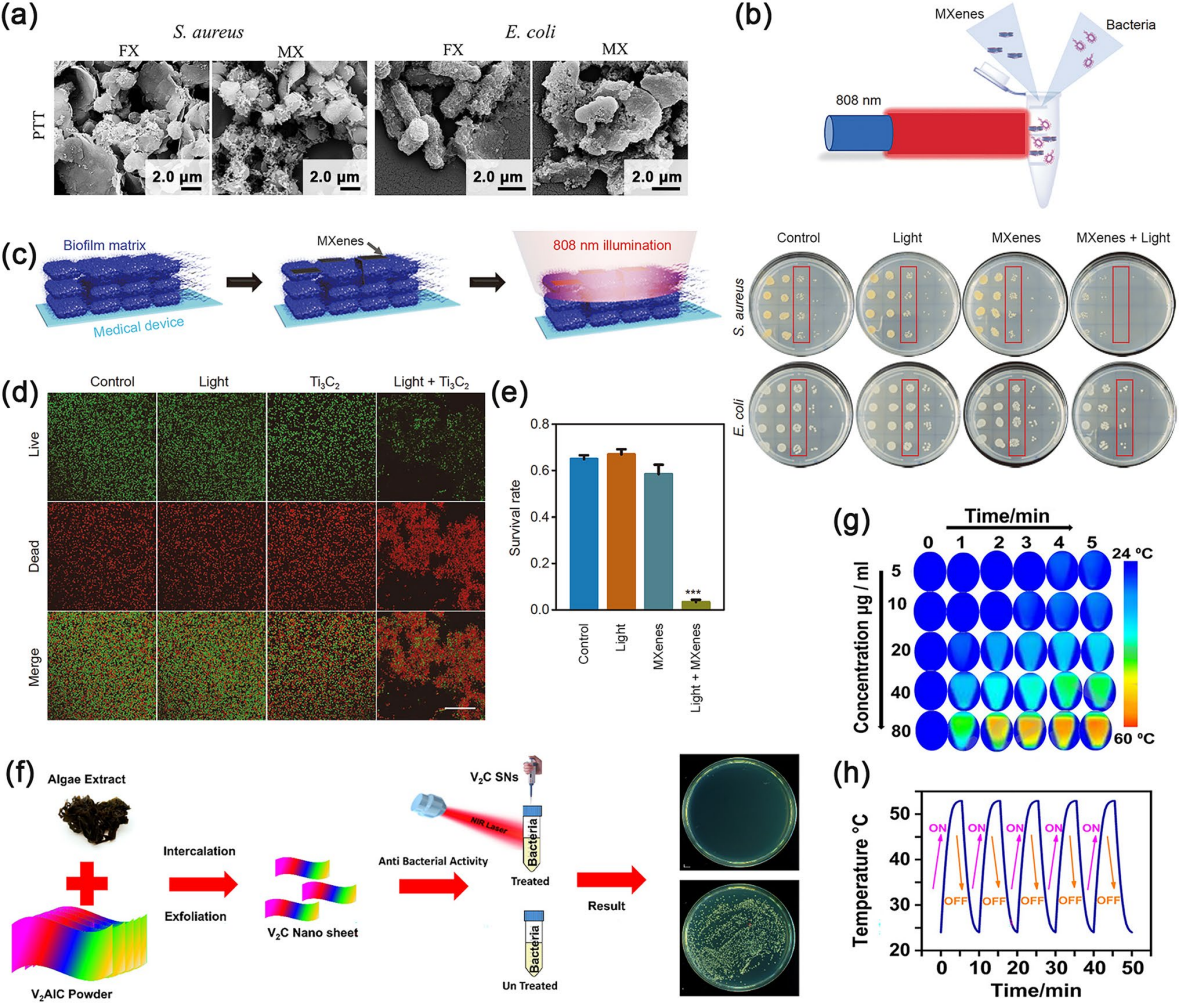
**a** SEM images of *S. aureus* and *E. coli* after treatments with few-layered Ti_3_C_2_T_x_ (FX) and multilayered Ti_3_C_2_T_x_ (MX) along with PTT [78]. Copyright 2021, Elsevier. **b** Colony-forming units images of *S.* *aureus* and *E.* *coli* without and with 20 min light treatments; **c** Schematic illustration of bacterial biofilms treated with MXenes and NIR light; **d** Fluorescence images of bacterial biofilms treated with MXenes and NIR light; **e** Survival statistics of bacterial biofilms treated with MXenes and NIR light [37]. Copyright 2021, Springer Nature. **f** Synthesis of V_2_C and its synergistic photothermal antibacterial effect; **g** Thermic photograph of V_2_C under NIR light; **h** Heating and cooling cycles of 40 μg mL^−1^ V_2_C under NIR light [142]. Copyright 2021, American Chemical Society

Owing to their extremely high photothermal conversion ability, outstanding biocompatibility, fascinating antibacterial properties and low cytotoxicity, MXenes have attracted extraordinary attention for biomedical applications [141]. For instance, as one of the earliest and most widely used MXenes, Ti_3_C_2_T_x_ exhibited the feature of high efficacy, small dosage, and fast function in antibacterial applications. According to Rosenkranz et al. [78], the use of few-layered Ti_3_C_2_T_x_ (FX) and multilayered Ti_3_C_2_T_x_ (MX) nanosheets as antibacterial PTT against *E. coli* and *S. aureus* was feasible. The biocompatibility experiment showed that certain eukaryotic cell lines were less cytotoxic when exposed to few-layered Ti_3_C_2_T_x_ nanosheets. And for the bacteria treated with few-layered Ti_3_C_2_T_x_, the damage of cell membrane and the loss of contents were more serious than the multilayered Ti_3_C_2_T_x_ group (Fig. 8a) [78]. With the effect of PTT, Ti_3_C_2_T_x_ destroyed the protective membrane and further inactivated the inherent bioactive matrix promptly. Upon 808 nm NIR light irradiation, significant antibacterial effects could be observed in a suspension of Ti_3_C_2_T_x_ nanosheets after only 20 min (Fig. 8b) [37]. Ti_3_C_2_T_x_ nanosheets have been found to show an obvious killing effect on a variety of bacteria, including methicillin-resistant *Staphylococcus aureus* and vancomycin-resistant *Enterococci* [37]. Furthermore, the rapid antibacterial strategy could suppress methicillin-resistant biofilms by destroying their structures as well as erasing the bacteria within them (Fig. 8c). The survival rate of bacteria in the biofilm of the experimental group was reduced by 95% compared to the control (Fig. 8d, e). Similar to Ti_3_C_2_T_x_, V_2_C displayed high structural stability and strong NIR absorption properties and has also been reported for photothermal ablation of bacteria. Zada and colleagues developed an algae extract-based controllable and green delamination approach to exfoliate V_2_C nanosheets with antimicrobial activity (Fig. 8f) [142]. The thermal images showed that the temperature of 80 μg mL^−1^ V_2_C nanosheets could reach above 50 ℃ within 5 min under the irradiation of 808 nm NIR light, which exceeded the tolerance level of *E. coli* and *B. subtilis* (Fig. 8g). In the laser on/off cycles (five times off and on), the temperature changes of V_2_C nanosheets suspension showed a negligible fluctuation and an insignificant decline, indicating satisfying photothermal stability of V_2_C nanosheets. Consequently, this suspension achieved an antibacterial efficiency of over 99.5% using V_2_C nanosheets with reliable photothermal properties.

#### Composites of MXenes and Other Antimicrobial Agents

**Fig. 9:**
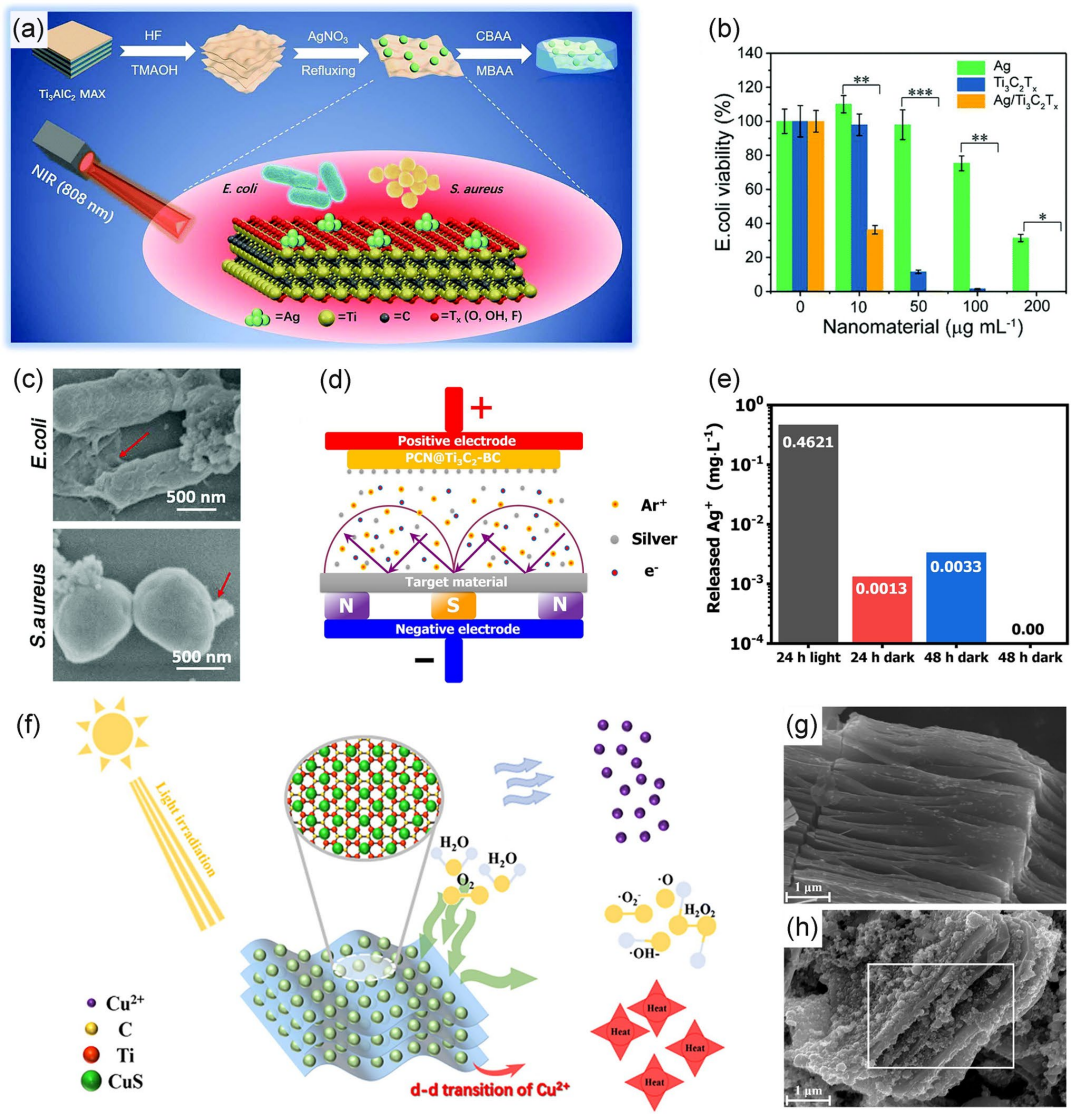
**a** Schematic illustration of photothermal antibacterial properties of Ag/Ti_3_C_2_T_x_; **b** Relative bacteria viability with NIR light irradiation after incubation with different concentrations; **c** HRTEM images of *E. coli* and *S. aureus* were treated with Ag/Ti_3_C_2_T_x_ under NIR light [41]. Copyright 2020, Royal Society of Chemistry. **d** Schematic illustration of the film loaded with Ag^+^; **e** Schematic illustration of the release of Ag^+^ under different treatments [143]. Copyright 2021, Elsevier. **f** Schematic illustration of antibacterial mechanism of Ti_3_C_2_T_x_/CuS; **g** SEM image of multilayered Ti_3_C_2_T_x_; **h** SEM image of Ti_3_C_2_T_x_/CuS [139]. Copyright 2021, Elsevier

The combination of MXenes with other antimicrobial agents offers a promising way to enhance the ability to inhibit bacteria reproduction. Nanoscale silver is an encouraging antimicrobial agent because of its broad spectrum and long-lasting antibacterial activity. It has been proven a promising strategy by decorating silver nanoparticles on the surface of 2D MXenes to achieve desired sterilization performance. Zhu et al. [41] reported the representative case study on utilizing Ag/Ti_3_C_2_T_x_ for synergistic antibacterial effect (Fig. 9a). In this work, silver ions were firstly adsorbed and then reduced by sodium citrate on negatively charged Ti_3_C_2_T_x_ nanosheets producing the Ag/Ti_3_C_2_T_x_ composites. Ag/Ti_3_C_2_T_x_ suspension of 200 μg mL^−1^ showed inferior antibacterial activity in the dark, but the composites could effectively kill all bacteria when exposed to 808 nm NIR light irradiation (Fig. 9b). The SEM results were also in line with the antibacterial tests, where the Ag/Ti_3_C_2_T_x_ under NIR irradiation showed palpable synergistic antibacterial performance. In the high-resolution image, it was evident that bacteria exposed to light underwent cytoplasm leakage and cytolysis (Fig. 9c). The antimicrobial efficacy of silver nanoparticles is associated primarily with Ag^+^ ions release. In the work of Nie et al. [143], they soaked the thin film containing Ti_3_C_2_T_x_/Ag in a certain volume of solution and irradiated it with a Xe lamp (500 W, 31.45 W cm^−2^). According to Fig. 9d, e, under light illumination, the concentration of released silver ions was 0.46 mg mL^−1^, almost 300 times relative to the control group. It indicated that light promoted the release of Ag ions, and this process resulted in enhanced antimicrobial efficiency.

CuS is also a widely recognized antibacterial agent with excellent light absorption properties and Cu^2+^ release ability. However, the incorporation of CuS into MXenes by simple physical mixing makes it difficult to achieve a controlled release of Cu^2+^, and excessive local accumulation may cause negative effects, ultimately leading to poor antibacterial activity. Recent studies indicated that MXenes are ideal support materials for fabricating nanohybrids and the surface decoration of CuS nanoparticles on them through in situ growth has emerged as an efficient method. In a recent study, Li et al. [139] prepared NIR responsive MXenes/CuS composites through the reaction between C_2_H_5_NS and Cu^2+^ adsorbed on the surface of multilayered Ti_3_C_2_T_x_, achieving the controllable release of Cu^2+^ could be achieved (Fig. 9f). As shown in the SEM images, CuS nanoparticles with diameters ranging from 100 to 500 nm were uniformly grown on the multilayered Ti_3_C_2_T_x_ surface (Fig. 9g, h). Under the irradiation of NIR light, the release of Cu^2+^ was significantly enhanced. Owing to the synergistic contributions from the photothermal effects of MXenes and CuS and the enhanced release of bactericidal Cu^2+^ ions, the antibacterial efficiency of the MXenes/CuS group exceeded 99% against both *E. coli* and *S. aureus*. Such results underscored the importance of the simultaneous action of hyperthermia and antibacterial metal ions, revealing the great potential of synergistic therapy.

#### MXenes-Based Photodynamic Hybrids

**Fig. 10:**
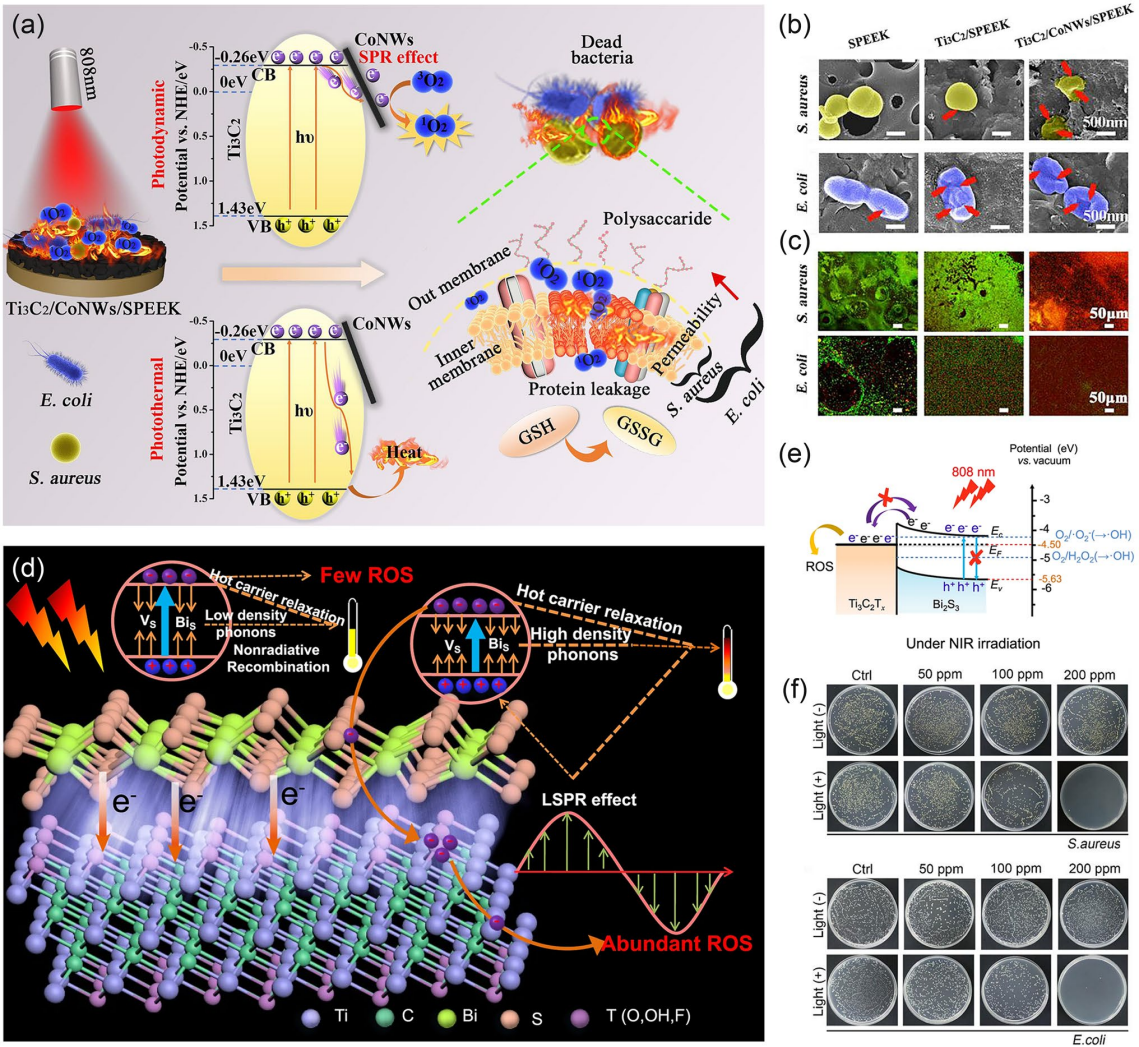
**a** Schematic illustration of photothermal/photodynamic antibacterial property of MXenes/Co; **b** TEM images of *S.* *aureus* and *E.* *coli* were treated with NIR light; **c** Confocal images of *S.* *aureus* and *E.* *coli* were treated with NIR light [39]. Copyright 2020, Elsevier. **d** Schematic illustration of photodynamic and photothermal mechanism between Ti_3_C_2_T_x_ and Bi_2_S_3;_ **e** The mechanism of the enhanced yield of ROS via NIR-induced progress is based on the Schottky heterostructure; **f** Colony-forming units images of *S. aureus* and *E.* *coli* without and with 10 min NIR light treatments [16]. Copyright 2021, Springer Nature

As photodynamic antibacterial strategies receive considerable research interest, MXenes-based hybrid systems are extensively investigated for their potential in ROS generation [16, 144]. However, the narrow bandgap of the Ti_3_C_2_T_x_ nanosheets (about 1.69 eV) severely hampers their performances due to the fast recombination of photo-generated electron-holes, which results in the limited release of ROS [51]. Previous research indicated that some photosensitive nanomaterials such as metal nanoparticles, metal oxides, carbon-based nanomaterials, and metal chalcogenides had shown their prospects in bacteria-killing under light irradiation [10]. However, the photo-induced ROS production on these materials is heavily impaired once without the cocatalysts, which usually act as electron traps and reactive sites. Accordingly, the establishment of heterojunctions between these photosensitive materials with metallic MXenes is considered an effective modified strategy for significantly separating photo-generated electron–hole pairs and thus promoting ROS production [47]. Many groups have verified such strategy to achieve the desired antibacterial performance based on photo-induced approaches. As depicted in Fig. 10a a 2D/1D heterojunction between Ti_3_C_2_T_x_ and cobalt nanowires has been presented, and the composites exhibited NIR-triggered photothermal and photodynamic synergistic antibacterial activity [39]. The electrons were excited from the valence band (VB) to the conduction band (CB) of Ti_3_C_2_T_x_ with NIR laser irradiation and transferred quickly to the surface of cobalt nanowires. In this way, electrons gathered on the surface of the cobalt nanowires and holes accumulated on the VB of Ti_3_C_2_T_x_, which hindered the recombination of electrons and holes. The adsorbed oxygen captured photogenerated electrons to generate ROS, leading to the diffusion of ROS from the heterojunction to the whole system. In comparison with the control group, the MXenes/Co group caused obvious bacterial lysis, as confirmed by SEM and confocal images (Fig. 10b, c). Analogously, Li et al. [16] designed Ti_3_C_2_T_x_/Bi_2_S_3_ composites and found that the Schottky barrier at the interfaces forcefully enhanced the amount of generated ROS (Fig. 10d, e). As a result, the composites could feasibly kill 99.92% of *E. coli* and 99.86% of *S. aureus* under 808 nm NIR irradiation within 10 min (Fig. 10f). It can be seen that the construction of heterojunctions provides new strategies for designing light-triggered devices for antibacterial applications. Here, the possibility that MXenes can be combined with a variety of nanomaterials opened up endless possibilities for such designs. To sum up, through the ingenious preparation of MXenes-based nanomaterials and subsequent appropriate modification, various photothermal-derived platforms could exhibit satisfactory in vitro bactericidal properties. We thoroughly investigated the literature database and summarized the advances in this topic by sorting their main components, antibacterial mechanisms, effects, and conditions, as shown in Table 3.

**Table 3:** Summary of in vitro photothermal-derived antibacterial applications using MXenes-based materials

Main components	Antibacterial mechanisms	Light source	Concentration (μg mL^−1^)	Treatment time (min)	Bacteria	Effect	References
Few-layered Ti_3_C_2_T_x_	PTT	808 nm, 5.7 W cm^−2^	100	5	*E. coli*	> 85%	[78]
					*S. aureus*	> 95%	
Ti_3_C_2_T_x_	PTT	808 nm, 0.4 W cm^−2^	100	20	*E. coli*	> 85%	[37]
					*S. aureus*	100%	
Ti_3_C_2_T_x_/Ag	PTT/Ag^+^ release	808 nm, 1.5 W cm^−2^	100	15	*E. coli*	100%	[41]
Ti_3_C_2_T_x_/Ag	ROS generation/Ag^+^ release	808 nm, 0.4 W cm^−2^	1.56	30	*E. coli*	100%	[145]
			3.12		*S. aureus*	100%	
Ti_3_C_2_T_x_/Ag	PTT/ROS generation/Ag^+^ release	Xe lamp, 31.45 W cm^−2^, 15 cm	–	30	*E. coli*	100%	[143]
					*S. aureus*	100%	
Ti_3_C_2_T_x_/Ag/Cu_2_O	ROS generation/Ag^+^ release	T5 energy saving lamp	2000	720	*P. aeruginosa*	99.7%	[48]
					*S. aureus*	99.7%	
Ti_3_C_2_T_x_/Au	PTT/ROS generation	808 nm, 2.0 W cm^−2^	160	8	*E. coli*	99.3%	[28]
					*B. subtilis*	100%	
Ti_3_C_2_T_x_/Co	PTT/ROS generation	808 nm, 1.5 W cm^−2^	–	20	*E. coli*	92.7%	[39]
					*S. aureus*	80.1%	
Ti_3_C_2_T_x_/Bi_2_S_3_	PTT/ROS generation	808 nm, 0.7 W cm^−2^	200	10	*E. coli*	99.9%	[16]
					*S. aureus*	99.9%	
Ti_3_C_2_T_x_/CuS	PTT/ROS generation/Cu^2+^ release	808 nm, 1.5 W cm^−2^	500	720	*E. coli*	99.6%	[139]
					*S. aureus*	99.1%	
Ti_3_C_2_T_x_/AgS	PTT/ROS generation	808 nm, 0.67 W cm^−2^	500	20	*S. aureus*	99.9%	[17]
Ti_3_C_2_T_x_/Cu_2_O	ROS generation/Cu^2+^ release	–	40	1200	*S. aureus*	95.6%	[144]
					*P. aeruginosa*	97.0%	
Ti_3_C_2_T_x_/TiO_2_	ROS generation	White light	100	180	*E. coli*	97.4%	[140]
TiVCT_x_	Physical damage/PTT	808 nm, 0.1 W cm^−2^	40	30	*E. coli*	100%	[146]
					*B. subtilis*	100%	
V_2_C	Physical damage/PTT	808 nm, 0.1 W cm^−2^	40	5	*E. coli*	99.5%	[142]
					*B. subtilis*	99.5%	

### In Vivo Antibacterial Applications

**Fig. 11:**
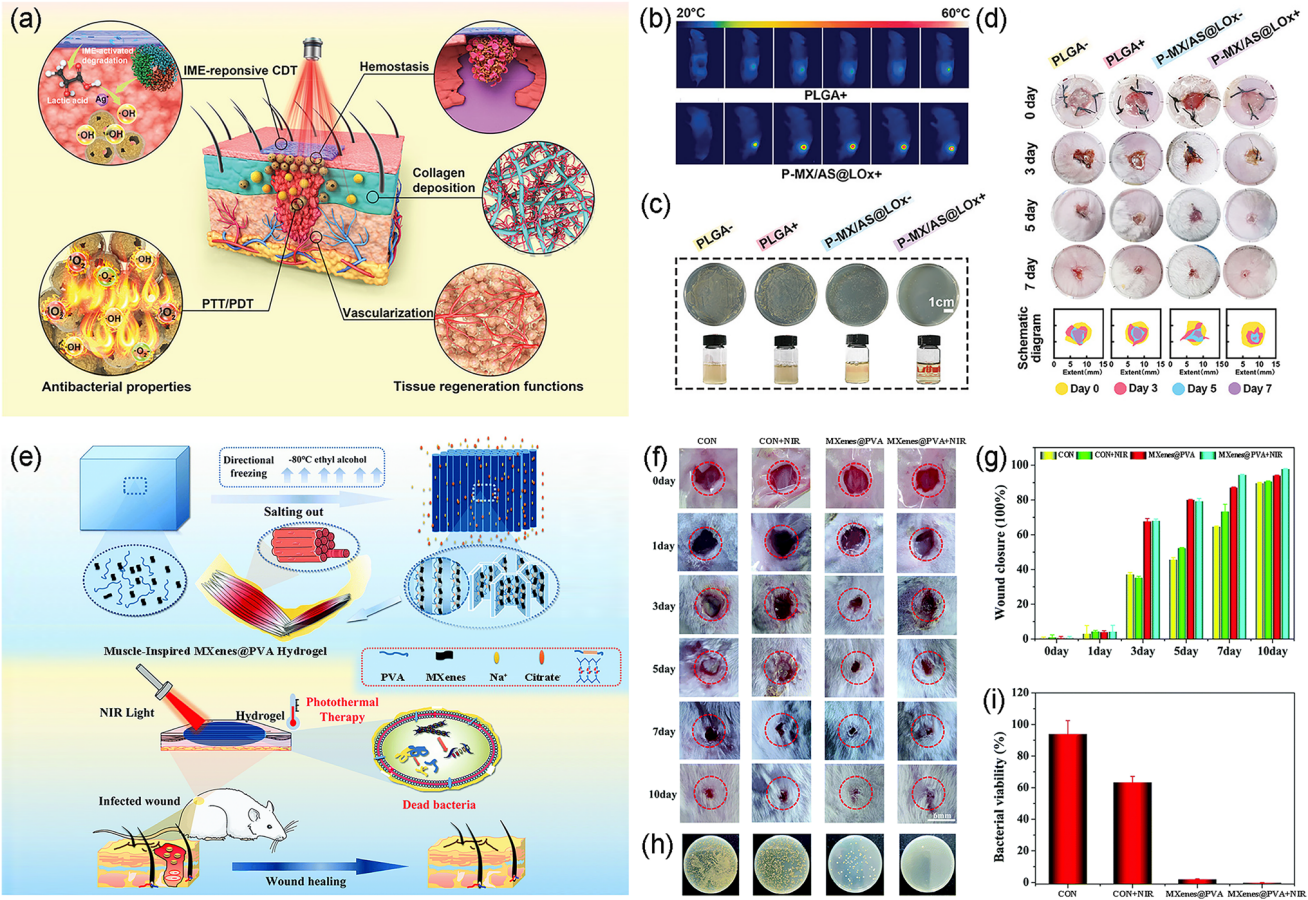
**a** Schematic illustration of the antimicrobial and infected wound repair effects by nanocatalytic membrane; **b** Surface NIR images of membranes under NIR light irradiation for 10 min; **c** Colony-forming units images of the bacterial colonies after different treatments; **d** Photographs of *S. aureus*-infected wounds healing after treatments [22]. Copyright 2021, Wiley–VCH. **e** Schematic illustration of the antimicrobial and infected wound repair effects by MXenes@PVA hydrogel; **f** Photographs of healing of *S. aureus*-infected wounds in different treatment groups; **g** Wound contraction evaluations of healing of *S. aureus*-infected wounds in different treatment groups; **h** Colony-forming units images of the bacterial colonies different treatments; **i** Quantitative bacterial viability based on **h** [148]. Copyright 2022, Royal Society of Chemistry

Skin, serving as the largest organ and the first line of body defense, maintains stability in the interior environment when faced with external threats [147]. Nonetheless, infected dermis wounds may cause pain, amputations or even death and have emerged as one of the severest threats to global health security [27]. Sponge, bandages, and gauze are the most routinely used wound dressings, and they possess ordinary functions by forming a physical parclose or assimilating exudate. However, they are unable to perform the biochemical action of eliminating bacteria and promoting healing. Consequently, designing newfangled light-triggered wound dressings that can promote bacteriostasis, enhance granulation tissue formation and accelerate cutaneous regeneration is desirable. To remedy bacteria-invaded stalled full-thickness wounds (Fig. 11a), Zhou et al. [22] group devised a nanoscale catalytic membrane (P-MX/AS@LOx) consisting of electrospun poly (lactic-co-glycolic acid) (PLGA) scaffolds, MXenes/Ag_2_S (MX/AS) bio-heterojunctions, and lactate oxidase (LOx). The MXenes/Ag_2_S bio-heterojunctions in the membrane not only exerted a mild photothermal effect and generated ROS under NIR light but also overwhelmed the hydroxyl radicals through Fenton-like reactions, which resulted in highly efficient synergistic sterilization. Upon exposure to NIR light, the temperature of the experimental group increased from 22.5 to 61.2 °C within 10 min, confirming the extraordinary efficiency of in vivo photothermal conversion (Fig. 11b). As shown in Fig. 11c, d, the nanoscale catalytic membrane remodeled stagnant chronic wounds into regenerative wounds by killing bacteria, stopping bleeding, promoting angiogenesis, boosting collagen deposition and enhancing epithelialization.

Aside from membrane, hydrogels are also recognized as a new type of wound dressing because of their high water absorbing abilities and porous structure, showing promising potentials in promoting wound healing. Inspired by the hierarchical assembly of anisotropic structures across multiple length scales of muscles, Li et al. [148] designed an anisotropic MXenes@polyvinyl alcohol (MXenes@PVA) hydrogel using a directional freezing-assisted salting-out method (Fig. 11e). Except for the excellent mechanical properties (stress up to 0.5 MPa, strain up to 800%), the hydrogel could also be used for local hyperthermia treatments at infected sites under 808 nm NIR irradiation. They employed a full-thickness *S. aureus*-infected wound model to evaluate the efficiency of MXenes@PVA hydrogel to cure infected skin wounds. As shown in Fig. 11f, g, the wound healing rate of the MXenes@PVA plus NIR group reached 98% after ten-day treatments, and most of the wounds were covered by new skin. The antibacterial activity of the experimental group was significantly higher than those of the control group, indicating that under NIR irradiation, MXenes@PVA hydrogel was effective in combating bacterial infection caused by *S. aureus* (Fig. 11h, i). MXenes@PVA hydrogel exhibited high toughness, anisotropy, and antimicrobial properties, hinting it could be an attractive dressing for antibacterial wound healing. In addition to the above composites, Ti_3_C_2_T_x_/Ag_3_PO_4_ [27], Ti_3_C_2_T_x_/MoS_2_ [149], Ti_3_C_2_T_x_/Bi_2_S_3_ [16], Ti_3_C_2_T_x_/Ag [41], and Nb_2_C [150] have all shown promising prospects for in vivo wound healing; the research progress could be found in Table 4.

**Table 4:** Summary of in vivo photothermal-derived antibacterial applications using MXenes-based materials

Main components	Dressing strategy	Antibacterial mechanisms	Bacteria	Light source (808 nm)	Treatment time (min)	Wound temp. (°C)	References
Ti_3_C_2_T_x_/Ag_3_PO_4_	Fibrous membrane	PTT/ROS generation/Ag^+^ release	*S. aureus*	1.5 W cm^−2^	10	58	[27]
Ti_3_C_2_T_x_/Ag_2_S/Lactate oxidase	Nanocatalytic membrane	Chemodynamic therapy/ PTT/ROS generation/Ag^+^ release	*S. aureus*	1.5 W cm^−2^	10	61.2	[22]
Ti_3_C_2_T_x_/MoS_2_	2D bio-heterojunction	PTT/ROS generation/Mo^4+^ release	*S. aureus*	1.5 W cm^−2^	5	57.0	[149]
Ti_3_C_2_T_x_	Colloidal solution	PTT	*S. aureus*	1.28 W cm^−2^	5	–	[151]
Ti_3_C_2_T_x_/PVA	Hydrogel	PTT	*S. aureus*	1.5 W cm^−2^	10	–	[148]
Ti_3_C_2_T_x_/Bi_2_S_3_	Wound dressing	PTT/ROS generation	*S. aureus*	0.7 W cm^−2^	10	–	[16]
Ti_3_C_2_T_x_/Ag	Hydrogel	PTT/Ag^+^ release	*S. aureus*	1.5 W cm^−2^	15	–	[41]
Nb_2_C/Titanium plate	Implant	PTT/ROS generation	*S. aureus*	0.7 W cm^−2^	10	52	[150]

### Antibacterial Water Evaporation and Purification

**Fig. 12:**
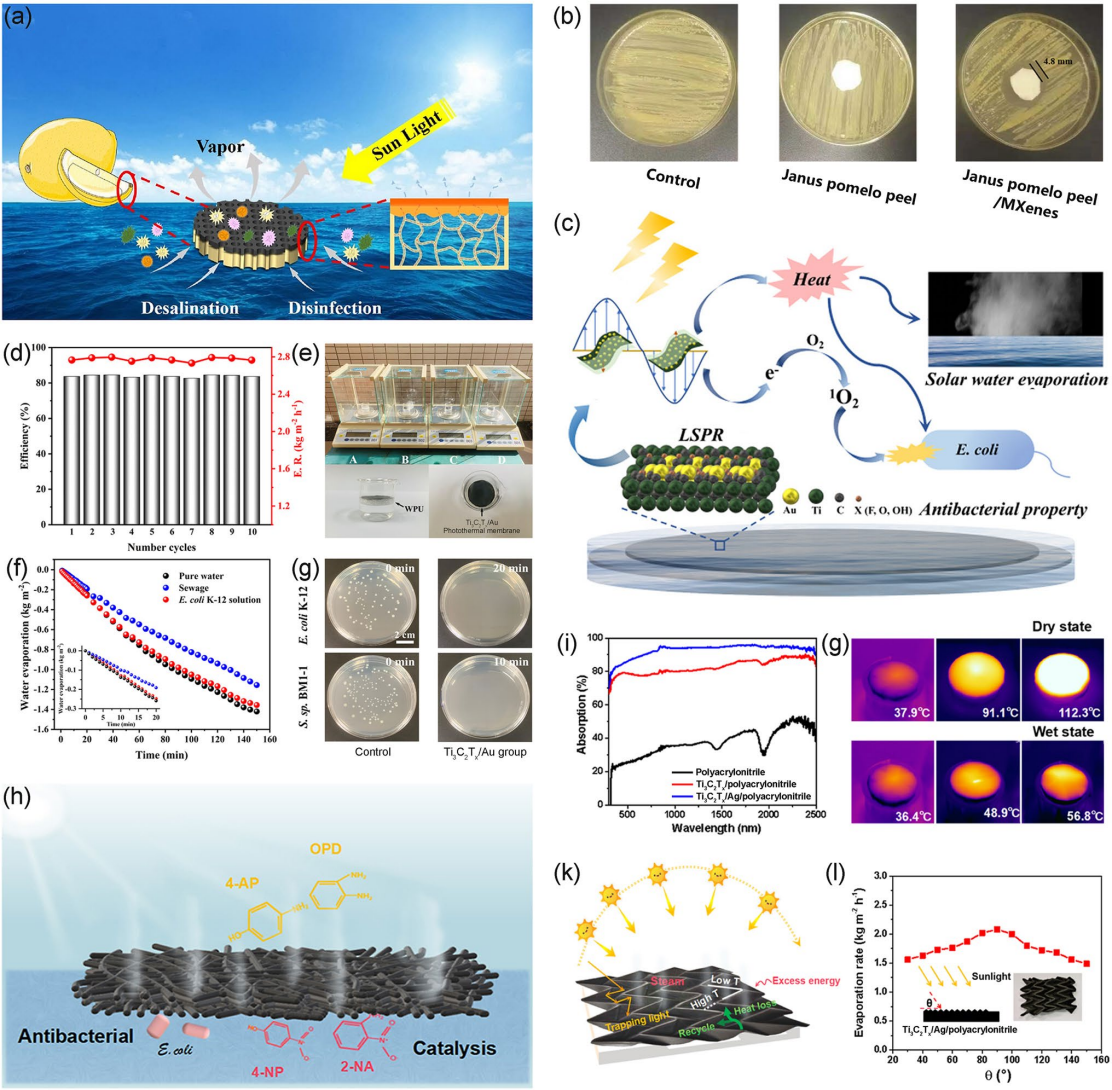
**a** Schematic illustration of water evaporation and antibacterial property of Janus pomelo peel/MXenes; **b** Photographs of diffusion inhibition zone tests [155]. Copyright 2021 Elsevier. **c** Schematic illustration of solar water evaporation and antibacterial property of Ti_3_C_2_T_x_/Au photothermal membrane; **d** Solar evaporation rate (right axis) and the corresponding evaporation efficiency (left axis) of Ti_3_C_2_T_x_/Au photothermal membrane over 10 cycles (each cycle sustained over 1 h); **e** Photograph of the evaporation devices (A: control group, B: pure water, C: sewage from the Pearl River, D: *E. coli* K-12 solution); **f** Water evaporation performance of different solution systems; **g** Colony-forming units images of different bacteria after treatment with Ti_3_C_2_T_x_/Au photothermal membrane under irradiation [42]. Copyright 2021, American Chemical Society. **h** Schematic illustration of water evaporation and antibacterial property of Ti_3_C_2_T_x_/Ag/polyacrylonitrile nanofiber membrane; **i** Ultraviolet–visible–near-infrared (UV–vis–NIR) absorption spectra in the wavelength range from 300 to 2500 nm; **g** IR thermal images in the dry and wet state, the left column is polyacrylonitrile nanofiber membrane, the middle column is Ti_3_C_2_T_x_/polyacrylonitrile nanofiber membrane, and the right column is Ti_3_C_2_T_x_/Ag/polyacrylonitrile nanofiber membrane; **k** Schematic illustration of evaporator under different incident angles of sunlight; **l** Evaporation rate of fabricated Ti_3_C_2_T_x_/Ag/polyacrylonitrile nanofiber-based origami evaporator under different incident angles of sunlight [44]. Copyright 2021, Elsevier

In recent years, solar-driven interfacial water evaporation has received increasing attention as a practical water purification solution for water-scarce regions [152, 153]. In comparison with traditional desalination technologies, solar evaporation systems demonstrate high-efficiency, low-cost, and eco-friendly performances [154]. MXenes with 2D nanosheet morphology and strong photo absorption capacity from UV to infrared (IR) range are excellent candidates for the fabrication of solar evaporators [152]. When these nanoscale photo-heaters are incorporated into film or hydrogel systems, they offer versatile potential to design antibacterial soft materials. Ideally, photothermal materials with antibacterial activity are more demanding for water purification applications as no extra antibiotics are necessary. Antibacterial properties of solar evaporators could mightily extend their service life by suppressing the formation of biofouling and inactivating pathogens in the water to ensure the production of fresh, clean and harmful microbial-free drinking water. Using Janus pomelo peel/MXenes as the photothermal center, Guan et al. [155] reported a low-cost and multifunctional steam generator with a hydrophobic top and hydrophilic bottom, ensuring efficient and stable water evaporation (Fig. 12a). It was found that there was no inhibition zone of *S. aureus* around Janus pomelo peel, while a bacteriostatic zone with a width of 4.8 mm is clearly observed around Janus pomelo peel/MXenes heterojunctions, indicating that Janus pomelo peel/MXenes composites possessed exceptional antibacterial activity during water evaporation (Fig. 12b). Furthermore, even after 50 cycles of simulated sunlight irradiation, a high-efficiency evaporation performance could be maintained. These interesting findings indicated that this cost-effective, environmentally friendly and sustainable photothermal sponge held the promise for large-scale wastewater treatment and purification. Besides the foam structure, Qu et al. [42] proposed a new strategy to construct a Ti_3_C_2_T_x_/Au photothermal membrane with efficient water evaporation capacity and antimicrobial activity under solar light irradiation (Fig. 12c). After ten cycles, the evaporation efficiency and rate of Ti_3_C_2_T_x_/Au membrane were still maintained at 83.63% and 2.664 kg m^−2^ h^−1^, respectively, displaying their recyclable potentials (Fig. 12d, e). By using the membrane, interfacial water evaporation was significantly enhanced compared to the control group (Fig. 12f). In dark, Ti_3_C_2_T_x_/Au membrane exhibited negligible antibacterial effect. In sharp contrast, it could effectively kill all bacteria under irradiation, and the authors attributed this phenomenon to the hot-zone formed at the air–water interface and enough sterilization time (Fig. 12g). In the antibacterial process, the primary ROS produced by Ti_3_C_2_T_x_/Au was detected as ^1^O_2_. The oxidative stress self-protection of bacterial cells was weakened by the presence of heat and ^1^O_2_. In addition to the design of a new efficient photothermal evaporation strategy based on surface-modified Ti_3_C_2_T_x_, this work also demonstrated the bactericidal potential of MXenes during water purification. Combining antibacterial agents with inherent bactericidal properties and MXenes into an integrated photothermal film is also a promising strategy. Liu et al. [44] reported a Ti_3_C_2_T_x_/Ag/polyacrylonitrile nanofiber-based evaporator, in which the flexibility and foldability of the nanofiber membrane were achieved by electrospinning technology (Fig. 12h). The combination of Ti_3_C_2_T_x_ and Ag nanoparticles resulted in broad-spectrum light absorption, efficient photothermal conversion, exceptional catalytic performance (Fig. 12i, j), as well as high antibacterial activity with 99.9% killing efficiency. This evaporator not only exhibited high structural flexibility, excellent performance, and multiple functions but also could achieve a maximum evaporation rate of 2.08 kg m^−2^ h^−1^ under one sun irradiation (Fig. 12k, l). In the long run, this flexible, biofouling-resistant and efficient nanofiber membrane may find practical application in wastewater treatment. To summarize, as shown in Table 5, MXenes-containing evaporators for solar-driven water evaporation and purification have exhibited various successful cases, shining light on practical applications in long-term water treatments.

**Table 5:** Summary of antibacterial applications using MXenes-containing evaporators

Main components	Evaporator strategy	Antibacterial mechanisms	Bacteria	Antibacterial effect	Water evaporation rate (kg m^–2^ h^–1^)	References
Ti_3_C_2_T_x_/Au/ polyvinylidene fluoride	A superior interface photothermal conversion material to achieve efficient evaporation and antibacterial properties	PTT/ROS generation	*E. coli* K-12	100%	2.66^*a*^	[42]
			*Spingopyxis* sp. BM1-1	100%		
Ti_3_C_2_T_x_/Ag/ polyacrylonitrile	A evaporator promising for practical clean water production	Physical damage/Ag^+^ release	*E. coli*	100%	2.08^*b*^	[44]
Ti_3_C_2_T_x_/cellulose	A flexible anti-biofouling fibrous photothermal membrane	Physical damage	*E. coli*	99.9%	1.44^*b*^	[152]
			*S.* *aureus*	99.9%		
Ti_3_C_2_T_x_/ dimethylsiloxane/ hydrophilic polylactic acid/TiO_2_	A flexible MXene-based Janus porous fibrous membranes for solar-driven desalination and antibacterial properties	Physical damage	*E.* *coli*	100%	1.0^*b*^	[156]

^*a*^2 sun illumination; ^*b*^1 sun illumination

### Flexible Antibacterial Textiles

**Fig. 13:**
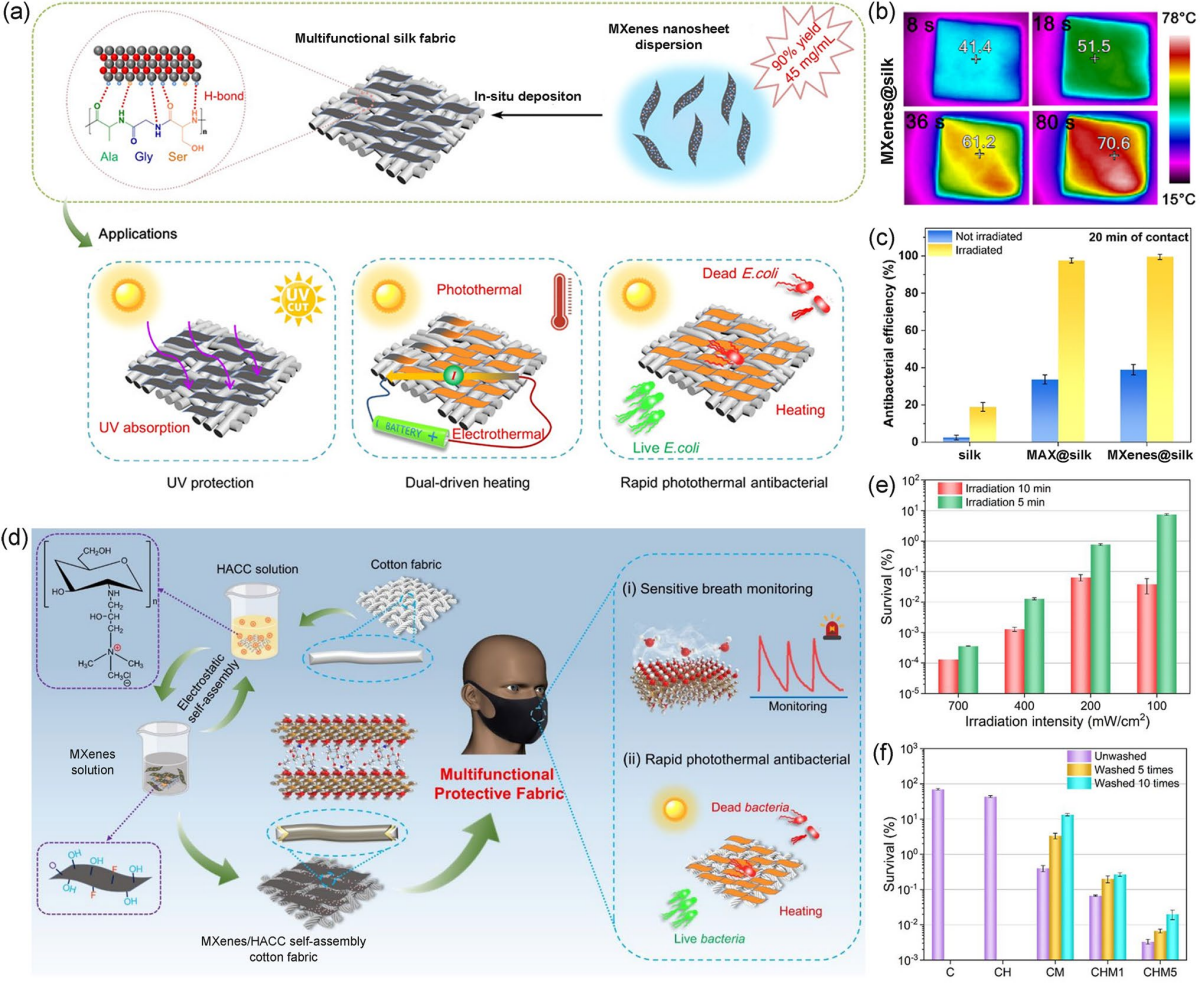
**a** Schematic illustration of preparation and derivative functions of MXenes-based multifunctional silk fabric; **b** IR thermal images of the MXenes@silk; **c** The antibacterial efficiency of the different samples contacted for 20 min [45]. Copyright 2021, American Chemical Society. **d** Schematic illustration of preparation and application of multifunctional protective MXenes/HACC cotton fabric; **e** Antibacterial activity with different irradiation intensity and time; **f** Inactivation of fabric samples against *E. coli* and *S. aureus* before and after washing [141]. Copyright 2022, American Chemical Society

Smart flexible electronic devices, such as wearable sensors, medical monitoring devices and soft robots, have raised extensive public interest [36, 43, 49]. As well as their comfort and skin-friendliness, textile materials are breathable and flexible, making them an excellent option for flexible wearable devices. Nevertheless, achieving the multifunctional properties while maintaining the intrinsic advantages of the fabrics is still challenging. Since MXenes with layered structures exhibit unique surface chemical properties and comparable electrical conductivity to metals, they have been widely applied for fabricating flexible smart fabrics through dip coating and spray coating approaches [157, 158]. Yan et al. [45] reported the preparation of MXenes-decorated silk fabric with satisfying UV protection performance, electrothermal conversion capability and photothermal antibacterial property through in-situ dip-coating Ti_3_C_2_T_x_ nanosheets onto silk fabric (Fig. 13a). Under continuous irradiation, the surface temperature of the fabric sample increased linearly, reached a saturation temperature within 100 s, and remained stable until the light was turned off (Fig. 13b). The rapid light response and stable photothermal conversion of this silk fabric also endowed it with high antibacterial capacity. Upon exposure to light irradiation for 20 min, the experimental group attained a 99.5% antibacterial efficiency (Fig. 13c). At the same time, the fabric of MXenes-containing silk still possessed skin-friendly properties, such as breathability and flexibility. Yan et al. [141] also designed a MXenes-based cotton fabric with micro breathing monitoring and rapid-photothermal antibacterial capabilities (Fig. 13d). Through electrostatic self-assembly, MXenes nanosheets and chitosan quaternary ammonium salt (HACC) strongly interacted while retaining the original breathability and softness of the cotton fabric. Based on the frequency and breathing depth of users, the cotton fabric could accurately monitor physiological health activities. This cotton fabric also showed promising photothermal conversion ability, washing resistance and cycling stability. Furthermore, the fabric showed an antibacterial efficiency of nearly 100% against *E. coli* and *S. aureus* within 5 min under irradiation with the intensity of 400 mW cm^−2^ (Fig. 13e). Moreover, the antibacterial efficiency against both bacteria was still higher than 99% after being washed for ten times, indicating broad-spectrum photothermal antibacterial ability and stable repeatability (Fig. 13f). Similarly, as summarized in Table 6, MXenes-based multifunctional fabrics such as wearable electronics, self-disinfecting textiles, anti-infection treatment platforms, and textile coating have been successfully practised, showing a promising application prospect.

**Table 6:** Summary of photothermal-derived antibacterial applications using MXenes-containing fabrics

Main components	Fabric strategy	Antibacterial mechanisms	Light source	Bacteria	Effect	References
Ti_3_C_2_T_x_/PDA^*a*^/Ni^2+^	A knittable and sewable spandex yarn with conductive nacre-mimetic composite coating	Physical damage/PTT	808 nm, 2.0 W cm^−2^	*E. coli*	99.9%	[46]
Ti_3_C_2_T_x_/PCN-224 MOFs^*b*^	A smart photothermochromic self-disinfecting textile material	PTT/ROS generation	Xe lamp, 500 W, λ ≥ 420 nm, 15 cm distance	*E. coli*	99.9%	[157]
				*S. aureus*	99.9%	
Ti_3_C_2_T_x_/Chitosan quaternary ammonium salt	A smart wearable multifunctional protective cotton fabric	PTT	Xe lamp, 0.4 W cm^−2^	*E. coli*	100%	[157]
				*S. aureus*	100%	
Ti_3_C_2_T_x_	A multifunctional heating fabric based on natural fiber	PTT	Xe lamp, 0.4 W cm^−2^	*E. coli*	99.6%	[45]
Ti_3_C_2_T_x_/Zeolite imidazole framework-8/Polylactic acid	A stimuli-responsive multifunctional electrospinning membrane	PTT/ROS generation	808 nm, 1.5 W cm^−2^	*E. coli*	100%	[159]
				Methicillin-resistant *Staphylococcus aureus*	100%	
Ti_3_C_2_T_x_/MoO_3–x_ quantum dots	A washable antibacterial polyester fabric	ROS generation	Sunlight	*E. coli*	~ 99%	[160]
				*S. aureus*	~ 99%	

^*a*^PDA: polydopamine; ^*b*^PCN-224 MOFs: porous coordination network metal–organic frameworks

## Conclusions and Perspectives

### Conclusions

As summarized in this review, the past decade witnessed rapid progress in unlocking the potential applications of MXenes-based materials in antibacterial and related fields. From the diverse synthetic strategies to microstructure examinations, we have gained basic knowledge of the nature of this family of alluring materials. Benefiting from their intrinsic photothermal or photodynamic ability, MXenes can be directly utilized as PTAs or PSs presenting great application potential in the rapid sterilization and disinfection. Hyperthermia generated by MXenes due to photo-to-thermal conversion could inactivate the bioactive matrix such as proteins and polysaccharides in microbial cells, causing the inactivation of pathogenic cells. Moreover, multitudinous active sites of MXenes make various modifications feasible; for instance, heterojunction construction could effectively postpone the recombination of electrons and holes, thus promoting ROS generation. To date, plentiful creative and elaborate designs have been implemented in the applications of antibacterial and related fields, indicating the thriving potential of MXenes in the post-antibiotic era.

### Challenges and Perspectives

Despite rigorous research and significant accomplishments so far, numerous fundamental issues remain unresolved. We have compiled a list of concerns that need to be addressed in order to push this field forward:

1. Primarily, from the standpoint of green and safe synthesis, the use of fluorine-containing etchants, which is present in the most commonly used preparation process, poses a significant risk to the environment and even to the health of researchers. And the subsequent treatment of waste liquid must meet stringent standards to fulfill the criterion for safe disposal handling. The novel preparation methods reported by using other fluorine-free reagents or reaction systems have received a lot of attention; however, it is not adaptable to a wide range of applications.

Therefore, the advanced preparation methods of MXenes are needed to realize practical applications. Experimentally, new etchants and intercalants, for a feasible MXene synthesis protocol, to achieve desired termination groups on the surface of MXenes and finely tuned properties are necessary to be explored.

(2) Since research into photothermal MXenes is still in its early stages, the mechanism of photo-to-thermal conversion is not fully understood. The absence of studies of interrelated issues makes the practical design of MXenes challenging.

Due to the chemical versatility of MXenes, more in-depth research and understanding of the photothermal mechanism is urgently needed, and developing a data pool of versatile MXenes, including full absorption spectra, temperature changes against optical power density, temperature evolution against time, the effects of synthetic protocols on photothermal performance and the general rules for optimizing photothermal performance will be beneficial.

(3) On account of the extremely high specific surface area, 2D materials with poor thermal stability are impressionable to the environment, among which MXenes are the sensitive ones in this category. MXenes can be readily decomposed to produce the oxides due to the synergistic contributions from air, moisture, and light. In practical applications, the stability of MXenes is of critical importance to ensure excellent photo-to-thermal conversion ability and effective antibacterial behaviors.

Thus, exploring and understanding the oxidation kinetics of MXenes is of great significance in predicting their changes in composition and performance over time. Besides, given that the oxidation of MXenes primitively occurs on the edges, passivating the edges via stable oxides or impervious materials can be considered.

(4) Photothermal-derived synergistic antibacterial therapy has emerged in the recent few decades, and the antibacterial mechanisms on MXenes need to be better established. Generally, an important attribute of bacterial inactivation is the damage to protective membrane structure which further leads to intracellular disturbance of homeostasis. There have been many efforts to verify the particular structure and processes whose alteration, under exposure to hyperthermia, causes bacteria death. Nonetheless, given the ambiguous and intricate interrelationships between the diverse structures and cellular functions, comprehension of experimental results is confused.

In this regard, systematic biological characterization is urgently needed to grasp the microscopic alteration of the critical component such as the outer and inner membrane, peptidoglycan cell wall, nucleoid, DNA, RNA, ribosomes, and diverse enzymes as well as the underlying structure-property relationships.

(5) Phototherapy itself still has several limitations, such as unitary treatment mode, local high power and high concentration requirements, and uncertain biocompatibility.

Hence, photothermal-derived synergistic strategies may need to be combined with other therapies, including but not limited to pharmacotherapy, chemotherapy, immunotherapy, starvation therapy and radiotherapy. For the selection of appropriate light power, imaging guidance is a befitting auxiliary to monitor the distribution of therapeutic agents to guide milder phototherapy.

(6) Another scruple is that biocompatibility is a pivotal issue in the therapeutic process. The cognition of the long-term effects on human health and the mechanisms of cytotoxicity is still insufficient. Although several remarkable achievements have been made in the in vivo treatments and short-term safety has been confirmed in these studies, more research and evaluations are essential to examine their long-term biosafety. Meanwhile, the potential impact of the degradation products is also necessary to be taken into consideration, which may lead to bioaccumulation and tissue toxicity.

To solve these issues, the computational simulation might be introduced to partly calculate the transformation of therapeutic agents and estimate the probable effects on healthy cells.
